# Structure of the 4-1BB/4-1BBL complex and distinct binding and functional properties of utomilumab and urelumab

**DOI:** 10.1038/s41467-018-07136-7

**Published:** 2018-11-08

**Authors:** S. Michael Chin, Christopher R. Kimberlin, Zygy Roe-Zurz, Pamela Zhang, Allison Xu, Sindy Liao-Chan, Debasish Sen, Andrew R. Nager, Nicole Schirle Oakdale, Colleen Brown, Feng Wang, Yuting Yang, Kevin Lindquist, Yik Andy Yeung, Shahram Salek-Ardakani, Javier Chaparro-Riggers

**Affiliations:** 1 Cancer Immunology Discovery, Pfizer Inc., 230 E. Grand Ave, South San Francisco, CA 94080 USA; 2 Department of Anesthesiology, University of Michigan, Ann Arbor, MI 48105 USA; 3 Present Address: Technion – Israel Institute of Technology, Haifa, 3200003 Israel; 4 Present Address: Bristol-Myers Squibb, 700 Bay Rd, Redwood City, CA 94063 USA

## Abstract

4-1BB (*CD137*, *TNFRSF9*) is an inducible costimulatory receptor expressed on activated T cells. Clinical trials of two agonist antibodies, utomilumab (PF-05082566) and urelumab (BMS-663513), are ongoing in multiple cancer indications, and both antibodies demonstrate distinct activities in the clinic. To understand these differences, we solved structures of the human 4-1BB/4-1BBL complex, the 4-1BBL trimer alone, and 4-1BB bound to utomilumab or urelumab. The 4-1BB/4-1BBL complex displays a unique interaction between receptor and ligand when compared with other TNF family members. Furthermore, our ligand-only structure differs from previously published data. Utomilumab, a ligand-blocking antibody, binds 4-1BB between CRDs 3 and 4. In contrast, urelumab binds 4-1BB CRD-1, away from the ligand binding site. Finally, cell-based assays demonstrate utomilumab is a milder agonist than urelumab. Collectively, our data provide a deeper understanding of the 4-1BB signaling complex, providing a template for future development of next generation 4-1BB targeted biologics.

## Abstract (web summary)

The costimulatory T-cell receptor 4-1BB is an immuno-oncology target. Here the authors present the ligand bound 4-1BB receptor crystal structure in addition to the structures of 4-1BB bound with two therapeutic antibodies and verify the antibody binding sites by mutational analysis, which is of interest for further 4-1BB therapeutics development.

## Introduction

In the last decade, immuno-oncology has emerged as one of the most promising therapeutic approaches to targeting cancer based on the potential for durable and complete disease remission^1^. This is built largely on the success of two checkpoint inhibitors, anti-CTLA4 (ipilimumab) and anti-PD1 (pembrolizumab), which have demonstrated remarkable efficacy both alone and in combination^1–3^. Excitement in the pharmaceutical industry is highlighted by the large number of clinical trials with these molecules: pembrolizumab alone is in over 600 clinical studies (June 2018, clinicaltrials.gov). However, thus far, these inhibitors are effective in only a fraction of patients treated^1,4,5^.

Many of the newer therapeutics moving forward in the clinic are agonistic antibodies that target costimulatory receptors in the tumor necrosis factor receptor superfamily (TNFRSF) such as 4-1BB, OX40, CD40, GITR, and CD27^6^. These agents contrast with ipilimumab and pembrolizumab, which are antagonistic antibodies affecting T-cell activation and exhaustion^7,8^. 4-1BB is not expressed on naive T cells but is rapidly upregulated after T-cell receptor engagement with cognate MHC:peptide complex expressed on antigen presenting cells^9–11^. Upon binding to 4-1BB ligand (4-1BBL or *TNFSF9*), 4-1BB signaling results in increased expression of pro-survival molecules via NF-κB signaling^12^. Initial studies demonstrated antitumor effects of agonistic 4-1BB antibodies with pronounced tumor regression in mastocytoma and sarcoma mouse models, and required both CD4^+^ and CD8^+^ T cells^13^. Subsequently, agonistic antibodies against 4-1BB were found to be effective in reducing or eliminating multiple tumors in murine models of melanoma, glioblastoma, lymphoma, renal cell carcinoma, and colon cancer among others^14–17^.

Based on these pre-clinical data, several companies have developed agonist 4-1BB antibodies. The two leading molecules in the clinic are utomilumab (PF-05082566) and urelumab (BMS-663513). Utomilumab is a ligand-blocking IgG2 antibody, and urelumab is a non-ligand-blocking IgG4 antibody^18,19^. Both isotypes are characterized by generally lower Fc*γ*R interaction, although IgG4 is known to engage with Fc*γ*RI and Fc*γ*RIIB more than IgG2^20^. In addition, while there have been anecdotal reports of differences in activity in 4-1BB signaling and induction of NF-κB, both antibodies enhance T-cell function and promote antitumor activity in vitro and in vivo^18,21–23^. However, despite the potential benefits of 4-1BB agonist antibody therapy, a recent integrated safety analysis of urelumab by Segal et al. documented toxicity in phase I and II clinical trials (NCT00309023, NCT00612664, NCT01471210) with grade IV hepatitis occurring in some patients at doses >1 mg kg^−1^ ^24^. Utomilumab shows reduced toxicity with fewer grade III–IV adverse effects and no dose-limiting toxicity reported for doses up to 10 mg kg^[−1 25–27^. Nonetheless, the differences in toxicity between the two antibodies are not fully understood, and could be due to differences in their agonist activity or other 4-1BB binding properties. In this work, we compare the binding epitopes and the mechanism of 4-1BBL blockade to examine the structural basis of the therapeutic efficacy of the two 4-1BB clinical antibodies.

4-1BB is a glycosylated type I membrane protein composed of four cysteine-rich pseudo repeats (CRDs) forming the extracellular domain, a short helical transmembrane domain, and a cytoplasmic signaling domain^28^. The extracellular domains of TNFRs range from one to four CRDs and typically form elongated structures. As such, antibodies targeting these molecules can bind in many modalities. The 4-1BB binding partner, 4-1BBL, is a type II membrane protein of the TNF superfamily^29,30^. Members of the TNFSF typically present as a homotrimeric complex, and are commonly expressed on cell membranes although some contain proteolytic processing sites that allow them to be released as soluble factors^31–33^. TNFSF members can be broadly categorized into three groups. Group 1 (conventional group) representative members include TNF, Apo2L/TRAIL, LTα, RANKL, LIGHT, and CD40L^34–36^. They adopt the canonical bell-shaped homotrimer structure most commonly associated with TNFSF members and bind their receptors similarly involving a conserved hydrophobic residue in the DE loop. Group 2 (EF-disulfide group) representative members include APRIL, BAFF, and EDA. The hallmark of this group is a disulfide bond between the E and F strands, and a more compact homotrimer structure. Group 3, including 4-1BBL, GITRL, and OX40L, is the most divergent group based on sequence. GITRL and OX40L structures have been reported, and adopt a flatter overall conformation^34,37^. Previous structural studies found that the homotrimer of 4-1BBL extracellular domain forms a unique extended pinwheel conformation not previously seen in other TNFSF members, suggesting a novel binding mode for this receptor–ligand complex^38^. We wanted to investigate how this unique structure might impact receptor engagement and interactions with therapeutic antibodies. Consequently, we solved a series of structures that help us understand the structure–function relationship of the 4-1BB/4-1BBL signaling complex and how therapeutic antibodies could perturb this system. Here, we present the structure of the hetero-hexameric 4-1BB/4-1BBL complex. Moreover, we have obtained the structure of the 4-1BBL homotrimer alone which is markedly different from previously published data^38^. Additionally, we determined the structures of utomilumab Fab and urelumab Fab bound to 4-1BB. Each of the antibodies bind to defined regions that are well separated both spatially and functionally on 4-1BB. Cell-based assays demonstrate distinct differences in activation by both antibodies. Finally, using confocal microscopy, we demonstrate that urelumab can induce 4-1BBL-dependent receptor clustering, distinguishing it from utomilumab.

## Results

### Structure of the 4-1BB/4-1BBL complex

We determined the structure of the 4-1BB/4-1BBL complex, showing the complete hetero-hexameric assembly with the ligand trimer engaging three copies of the receptor. The receptor–ligand complex crystallized with one complete copy of the complex per asymmetric unit, in agreement with SEC-MALS data and the resulting model was solved by molecular replacement and refined to a final resolution of 2.13 Å (Supplementary Figure 1, Supplementary Table 1).

**Fig. 1:**
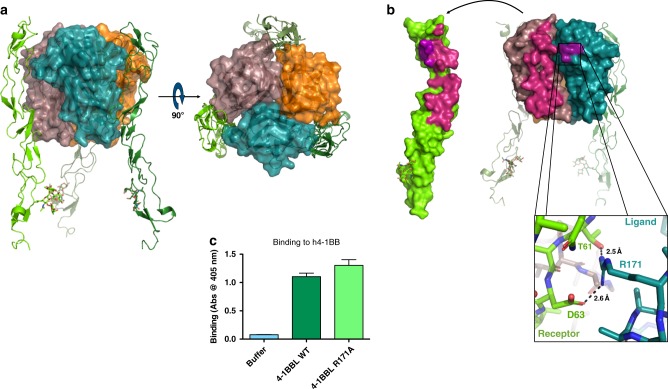
Structure of the 4-1BB/4-1BBL complex. **a** Side view of the 4-1BB/4-1BBL complex with ligand protomers shown in surface representation and colored orange, deep teal, and dirty violet for chains A, B, and C, respectively. Receptor molecules are shown in cartoon representation and colored smudge, forest, and chartreuse for chains X, Y, and Z, respectively. A 90° rotation shows the receptors evenly spaced around the exterior of the trimeric ligand. **b** Open book view of the binding interface between 4-1BB receptor and ligand highlighting the interactions between receptor chain Z and ligand chains C and B. Interactions between chain Z and chain C are shown in magenta while interactions between chains Z and chain B are shown in purple. Inset shows zoom in of interaction between adjacent ligand protomer B (deep teal) and the receptor chain Z (chartreuse). Arg171 of the ligand forms a salt bridge with Thr61 and Asp63 of the receptor and is the primary interaction between the receptor and the adjacent ligand protomer. **c** Biotinylated wild type or R171A mutant h4-1BB ligand was incubated with captured wild type h4-1BB receptor before washing and detection with streptavidin-HRP. Data shown as mean with s.d. (*N* = 3). Wild type (left, dark green) and R171A 4-1BBL (right, light green) bound to wild type 4-1BB at similar levels indicating that the R171 does not play a significant role in receptor engagement

The overall structure of the h4-1BB/4-1BBL complex shares many similarities with previously solved TNF receptor–ligand complexes. Namely, the ligand trimer adopts the canonical bell shape and the receptors are oriented parallel to the ligand trimer axis, binding along the exterior face of each ligand protomer (Fig. 1a). One significant difference, however, is that the receptor primarily engages with only a single ligand protomer rather than the cleft between adjacent ligand protomers (Fig. 1b). In all other examples of TNF receptor–ligand complexes, receptor binding is distributed relatively evenly between adjacent ligand molecules^34,39–44^. By contrast, in the 4-1BB/4-1BBL complex, an analysis with PISA shows that receptors bury an average of 823 Å^2^ of surface area on one ligand protomer, but only 121 Å^2^ of surface area on the adjacent ligand protomer (Table 1)^45^. This smaller adjacent interaction site occurs at the DE loop and is only clearly visible in receptor chain Z and ligand chain C of the three receptor–ligand pairs in the complex (Fig. 1b inset). 4-1BBL lacks the conserved hydrophobic residue found in the DE loop of conventional TNFSF ligands^34,35^. Here instead, R171 in the DE loop forms a salt bridge with D63 and a hydrogen bond with T61 of the receptor, comprising the majority of the interaction with that ligand protomer (Fig. 1b). That this interaction is only clearly seen in one of the three receptor–ligand pairs in the crystal structure suggests that this interaction may be transient or stabilized by crystal packing and not required for binding. Indeed, when we tested binding of 4-1BB to wild type or R171A mutant 4-1BBL by ELISA we saw no appreciable difference (Fig. 1c). Furthermore, when we measured affinities more precisely by surface plasmon resonance (SPR) experiments we saw only a minor difference in affinity with monovalent receptor binding to wild type 4-1BBL with a *K*_D_ of 680 ± 31 nM, while binding to R171A mutant 4-1BBL with a *K*_D_ of 521 ± 17 nM. These findings reinforce the idea that R171 does not play an important role in receptor binding and that each receptor primarily engages with a single ligand protomer (Supplementary Figure 2A, B, C).

**Table 1:** Ratio of receptor–ligand binding site areas

TNFRSF complex	PDB code	Receptor chain	Ligand chain	BSA (Å^2^)	Adjacent ligand chain	BSA (Å^2^)	Ratio of site 1 to site 2
4-1BB+4-1BBL		X	A	808	C	138	5.86:1
4-1BB+4-1BBL	6MGP	Y	B	827	A	114	7.25:1
4-1BB+4-1BBL		Z	C	833	B	112	7.43:1
CD40+CD154	3QD6	R	B	648	C	339	1.91:1
FasL+DcR3	4MSV	B	A	472	A^a^	460	1.02:1
LIGHT+DcR3	4J6G	D	B	545	B^a^	464	1.17:1
TNFα+TNFR2	3ALQ	R	A	716	B	619	1.16:1
OX40+OX40L	2HEV	R	F^a^	583	F	447	1.30:1
TNFβ+TNFR1	1TNR	R	A	599	A^a^	518	1.16:1
LTβ+LTβR	4MXW	R	B	490	D	332	1.48:1

^a^ denotes chain generated by symmetry operator

**Fig. 2:**
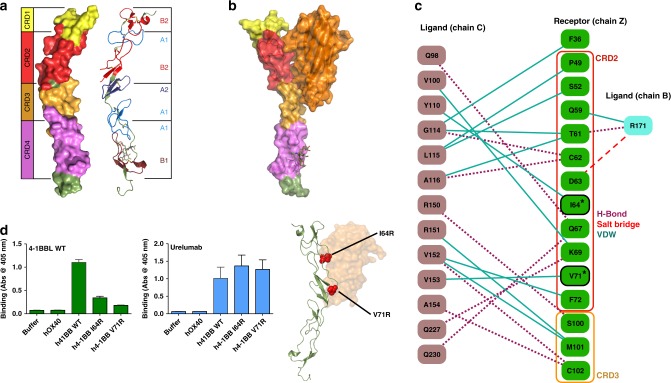
4-1BB CRD organization and interaction between receptor and ligand. **a** Breakdown of the CRD organization in the 4-1BB receptor ranging from CRD1 at the N-terminus to CRD4 at the C-terminus, highlighted on the left. Structural A and B motifs making up each individual CRD are highlighted on the right. **b** Side view of interaction between a single ligand protomer and receptor. 4-1BB ligand binds to CRD2 and CRD3 of the receptor. **c** Schematic diagram detailing the significant interactions between receptor chain Z (green) and ligand chains B (teal) and C (dark pink). Hydrogen bonds, salt-bridges, and van der Waals interactions are indicated in purple dashed lines, red dashed lines, and dark teal lines, respectively. Receptor residues mutated in D are outlined in black and marked with an asterisk. **d** Receptor mutants I64R and V71R were chosen to disrupt interaction with the ligand. Biotinylated wild type h4-1BB ligand was incubated with captured wild type or mutant h4-1BB receptor before washing and detection with streptavidin-HRP. Wild type ligand showed reduced binding to both I64R and V71R mutants relative to wild type receptor. Urelumab bound wild type and I64R and V71R mutant 4-1BB at similar levels indicating that mutant receptors were properly folded. Structure depicts position of receptor mutants. Data shown as mean with s.d. (*N* = 3). Receptor is shown in forest green cartoon with mutation sites highlighted in red spheres. Ligand (orange) is shown as a transparent surface

The structure of the 4-1BB/4-1BBL complex provided us with the opportunity to examine the architecture of the human 4-1BB receptor in detail (Fig. 2a). TNF receptors are composed of one to four CRDs with each CRD containing three disulfide bonds spread across two distinct structural modules (e.g., A1, B2, etc) as initially classified by Naismith and Sprang using sequence conservation and early TNFR structures^46^. Sequence analysis of the 4-1BB receptor suggested that it contained four CRDs with a somewhat unusual arrangement of A and B modules and this prediction is now confirmed in our crystal structures of human 4-1BB (Fig. 2a)^35^. At the N-terminus of 4-1BB, the partial CRD-1 is composed of just a single B2 module and only two disulfide bonds. CRD-2 shows the more typical arrangement with an A1 and B2 module and the full set of three disulfide bonds. CRDs 3 and 4 show less common arrangements with CRD-3 containing an A2 and A1 module while CRD-4 has only two disulfide bonds with an A1 and a B1 module.

Examination of the interaction between 4-1BB and 4-1BBL showed that the ligand binds along the entire length of receptor CRD-2 and the A2 motif of CRD-3 (Fig. 2b). The interface between the receptor and ligand is primarily mediated by hydrogen bonds and van der Waals interactions as detailed in Fig. 2c. The receptor-binding interface was confirmed by ELISA. 4-1BB mutations I64R and V71R showed reduced binding to wild type 4-1BBL (Fig. 2d). Additionally, these interactions were confirmed by SPR, again showing reduced binding for both I64R and V71R relative to wild type 4-1BB (Supplementary Figure 2D). This binding arrangement, involving CRDs 2 and 3, is similar to those seen in the DcR3:LIGHT, DcR3:FasL, TNFβ:TNFR1, and TNFα:TNFR2 receptor–ligand complexes^40–43^. Thus, h4-1BB uses a canonical receptor interface but engages ligand in a unique fashion, i.e., by binding primarily to a single 4-1BBL protomer rather than across the interface between two adjacent ligand protomers as seen in other examples of TNF receptor–ligand complexes as described in Table 1.

Furthermore, the structure of mouse 4-1BB was recently published^47^. Human and mouse 4-1BB show 64% sequence identity across the regions present in the crystal structures. However, when mouse 4-1BB is compared with human 4-1BB from the 4-1BB/4-1BBL complex, superposition of both receptors show significant structural similarity preserving all critical disulfide bonds amongst CRDs 1–4 and aligning with RMSD 0.7 Å^2^ (Supplementary Figure 3). One notable difference is in the glycosylation sites of the two receptors. Mouse 4-1BB contains two predicted N-linked glycosylation sites at N128 and N138, both of which show evidence of glycosylation in the crystal structures. Human 4-1BB lacks the N128 site and instead has a second glycosylation site at N149, with only this second site showing evidence of glycosylation in the structures of 4-1BB/4-1BBL complex and both 4-1BB–Fab complexes.

### Structure of 4-1BBL

One unexpected observation from our structure of the 4-1BB/4-1BBL complex was that the ligand was arranged as a canonical bell-shaped trimer, similar to conventional TNFSF ligands. This is in contrast to a previously solved structure of 4-1BBL which adopted a flattened pinwheel-like trimer (Supplementary Figure 4)^38^. Structures of other members of the divergent class of TNFSF-like OX40L and GITRL differ from the conventional TNFSF members with more compact, flattened trimer arrangements^34,37^. However, the previous structure of the 4-1BBL represents a significant departure even from these divergent members and it is unclear if this represents the native conformation of the 4-1BBL in the absence of receptor.

**Fig. 3:**
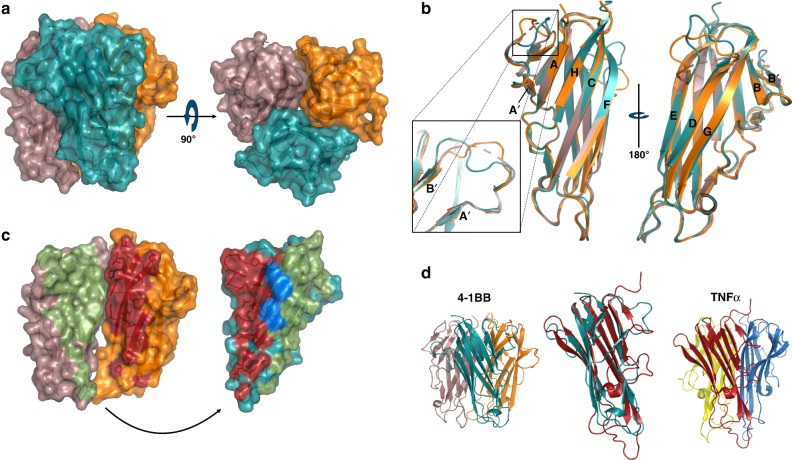
Structure of 4-1BBL. **a** Surface representation of assembled trimer seen from the side. A 90° rotation shows the threefold axis down the center of the trimer. **b** Superposition of all three ligand protomers in the asymmetric unit. Individual strands identified by black letters. Variability between protomers in DE loop seen in the outer beta sheet (right). Inset shows variability of loop A′B′ between ligand protomers. Ligand protomers shown in surface representation and colored orange, deep teal, and dirty violet for chains A, B, and C, respectively. **c** Open book view of the interfaces between ligand protomers. Interface between chain B and chain A highlighted in firebrick red. Interface between chain B and chain C highlighted in smudge green. Residues on chain B participating in both interfaces highlighted in marine. **d** Comparison of 4-1BBL (left panel) with TNFα (right panel, PDB code: 1TNF). Superposition of ligand protomers from 4-1BBL (teal) and TNFα (red)

To address this question directly we crystallized human 4-1BBL alone, determined the structure by molecular replacement, and refined to a resolution of 2.95 Å (Fig. 3, Supplementary Table 1). SEC-MALS data showed that soluble 4-1BBL was trimeric in solution (Supplementary Figure 1C). Concordantly, the asymmetric unit contained three 4-1BBL molecules in trimeric assembly, with residues 89–244 visible in the electron density (Supplementary Figure 5B). Importantly, the 4-1BBL trimer again formed the canonical bell shape seen in every other TNFSF structure to date excepting the pinwheel 4-1BBL structure (Fig. 3a). Much like previously solved structures of TNFSF ligands, 4-1BBL adopts the jelly-roll fold typical of the TNF homology domain, with the inner and outer sheets of the beta sandwich consisting of anti-parallel beta strands A′AHCF and B′BGDE, respectively (Fig. 3b)^48^. The three ligand protomers in the asymmetric unit can be readily superimposed with an average RMSD of 0.532 Å^2^ (Fig. 3b). While the structural jelly-roll fold of the protein core is well conserved between all three protomers, differences in conformation, discontinuous density and elevated B-factors can be seen in ligand loops A′B′ and DE suggesting a high degree of flexibility in the absence of a binding partner (Fig. 3b, inset). The inner sheet of each ligand protomer (AA′HCF) presents a largely hydrophobic face with the core of the trimer axis predominantly composed of alternating aromatic residues along strands C and F, reminiscent of the packing seen in conventional group ligands-like TNFα^48^. However, moving from the core trimer axis, out toward the EF and CD loops, the contacts at the protein–protein interface between adjacent protomers are more limited, thus sharing some characteristics with the divergent class TNFSF ligands. Analysis of binding interfaces with PISA shows that each protomer buries ∼1440 Å^2^ (Fig. 3c)^45^. The other divergent class members, GITRL and OX40L show a more splayed out arrangement relative to the central trimer axis while 4-1BBL protomers are oriented more vertically, reminiscent of members of the more conventional TNFSF classes such as TNFα (Fig. 3d, Supplementary Figure 6).

Comparison with our receptor-bound structure showed that the 4-1BBL trimer does in fact retain the overall bell-shaped arrangement in the absence of receptor (Supplementary Figure 7). Primary differences between the two structures occur in the loop regions. Specifically, the A′B′ loops in the ligand-only structure show varying degrees of disorder and variation between all three protomers. However, in the receptor–ligand complex the A′B′ loop from each ligand protomer adopts the same well-ordered conformation and can be clearly seen engaging the receptor. Additionally, in the ligand-only structure, only two of the three DE loops can be seen in the density, though with differing conformations. In the receptor–ligand complex only one of the three DE loops has well-ordered density where it forms a salt bridge with the adjacent receptor. Collectively, these structures show that 4-1BBL assembles much like the canonical TNFSF ligands and that the previously solved 4-1BBL structure may not represent the native conformation.

### Structures of clinical antibodies bound to 4-1BB

**Fig. 4:**
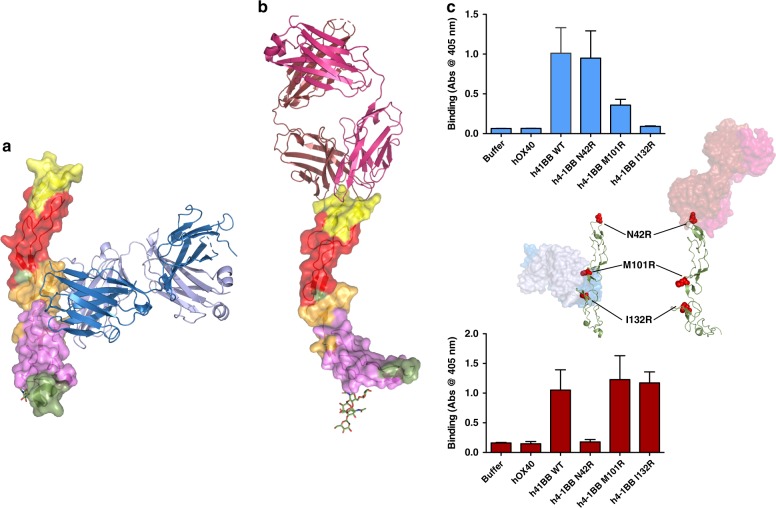
4-1BB bound to utomilumab and urelumab. **a** Utomilumab Fab bound to 4-1BB at the junction of CRD-3 and CRD-4. **b** Urelumab Fab bound to 4-1BB at the N-terminal portion of CRD-1. **c** Receptor mutants N42R or M101R and I132R were chosen to disrupt respective urelumab or utomilumab binding sites. Urelumab or utomilumab antibody was incubated with captured wild type or mutant h4-1BB receptor and subsequently washed before binding was detected by the addition of anti-human IgG Fc-HRP. Data shown as mean with s.d. (*N* = 3). As predicted by the antibody-bound structures, utomilumab shows reduced binding to M101R and I132R h4-1BB mutants but not N42R while urelumab shows the opposite trend. hOX40 was used as negative control. Structures depict position of receptor mutants. Receptors are shown in forest green cartoon with mutation sites highlighted in red spheres. Utomilumab (blue), urelumab (red) are shown in transparent surfaces

To understand the respective binding modes of utomilumab and urelumab, we crystallized complexes of 4-1BB receptor with Fabs of either antibody. Structures of 4-1BB/utomilumab Fab and 4-1BB/urelumab Fab complexes were determined by molecular replacement and refined to resolutions of 2.72 and 2.8 Å, respectively (Fig. 4a & b, Supplementary Table 1). Both structures contained two receptor-Fab complexes in the asymmetric unit with the interactions between the Fab variable domain and the receptors clearly visible in the electron density (Supplementary Figure 5C, D).

**Fig. 5:**
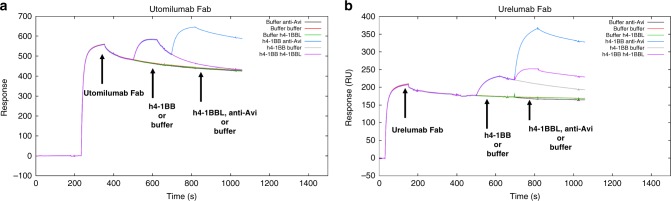
Sandwich ligand-blocking assay by SPR. Full sensorgrams for **a** utomilumab Fab was captured onto anti-human lambda surface or **b** urelumab Fab was captured onto anti-human kappa surface to determine blocking of the interaction of h4-1BB receptor with h4-1BBL. The legend shows the first analyte sample name followed by the second analyte. Triplicate measurements are consistent with one another; therefore, sensorgrams from third replicates are shown. Utomilumab Fab blocks the h4-1BB receptor/h4-1BBL interaction while urelumab Fab does not. Anti-Avi tag antibody was used to demonstrate binding to h4-1BB receptor by its Avi-tag when receptor is bound to utomilumab Fab or urelumab Fab

**Fig. 6:**
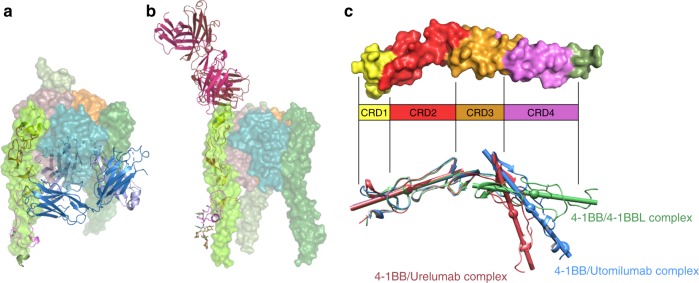
Superposition of the 4-1BB/4-1BBL complex with the antibody-bound structures and flexibility of human 4-1BB. **a** The structure of the utomilumab Fab bound to the 4-1BB receptor (cartoon representation) overlaid on the structure of the 4-1BB/4-1BBL complex (transparent surface representation) aligning on CRDs 1 and 2 of the receptor. Light chain of Utomilumab (light blue) clashes with the ligand. **b** The structure of the urelumab Fab bound to 4-1BB (cartoon representation) overlaid on the structure of the 4-1BB/4-1BBL complex (surface representation) aligning on CRDs 1 and 2 of the receptor. Urelumab binding to 4-1BB is compatible with ligand co-binding allowing for the formation of higher order aggregations. **c** Flexibility of the the human 4-1BB receptor. Receptor structures from the 4-1BB/4-1BBL complex, the 4-1BB–utomilumab complex, and the 4-1BB–urelumab complex were aligned on CRDs 1 and 2. Centroid axes for CRDs 1–2 or CRDs 3–4 were calculated using UCSF Chimera and displayed as rods. Both antibody-bound structures show a pivot point at the junction between the A2 and A1 motifs of CRD3 highlighting the significant range of motion possible in h4-1BB receptor

Comparison of the utomilumab- and urelumab-bound receptor structures showed that the antibodies have dramatically different binding sites, in terms of epitope and relative orientation of the antibody. Utomilumab binds along the side of 4-1BB, making contact at the junction between CRDs 3 and 4 (Fig. 4a). In contrast, urelumab binds to the very N-terminus of the 4-1BB receptor on CRD-1 (Fig. 4b). The utomilumab and urelumab binding sites on 4-1BB were confirmed by ELISA experiments in which 4-1BB mutants M101R and I132R showed reduced binding for utomilumab but not urelumab, while receptor mutant N42R showed reduced binding for urelumab but not utomilumab (Fig. 4c). SPR experiments measuring binding kinetics and affinities confirmed these results with utomilumab showing similar *K*_D_s for wild type and N42R h4-1BB (69 and 92 nM, respectively) but minimal binding for M101R and I132R mutants while urelumab had similar *K*_D_s for wild type, M101R, and I132R h4-1BB (22, 16, and 18 nM, respectively) but no appreciable binding for the N42R mutant (Supplementary Figure 8A & B). Additionally, the ligand-blocking activities were evaluated by SPR with a sandwich assay in which captured urelumab binds 4-1BB, which then subsequently binds 4-1BBL (Fig. 5b). In contrast, captured utomilumab was able to bind 4-1BB but the resulting complex could not subsequently bind 4-1BBL, presumably due to the steric occlusion (Fig. 5a). This result is further reinforced when examining the superposition of the antibody-bound receptors with the 4-1BB/4-1BBL complex (Fig. 6a, b).

Previous studies have shown that Galectin-9 plays a role in modulating 4-1BB signaling in mouse models and can bind to human 4-1BB through its sugar moieties in CRD4^47,49^. We measured binding of soluble monomeric HEK293-expressed human 4-1BB to Galectin-9 by SPR and found that it showed dose-dependent binding (Supplementary Figure 9A). We also evaluated whether either utomilumab or urelumab would disrupt this weak interaction with Galectin-9 by SPR comparing binding of 10 μM h4-1BB to 10 μM h4-1BB pre-mixed with saturating concentrations of either utomilumab Fab or urelumab Fab (Supplementary Figure 9B–D). In all cases, Galectin-9 binding was unperturbed, as might be expected given that both antibodies bind away from the sugar on CRD4.

Finally, when compared to the structure of the 4-1BB/4-1BBL complex, the structures of 4-1BB with either utomilumab Fab or urelumab Fab show a significant degree of flexibility within the receptor molecule. Namely, aligning receptors from both Fab complexes and the receptor–ligand complex along CRDs 1 and 2 showed a significant bend between the A2 and A1 motifs of CRD-3 in the urelumab-bound structure and a bend and rotation at the same position in the utomilumab bound structure (Fig. 6c).

### Activity and ligand-induced clustering of clinical antibodies

**Fig. 7:**
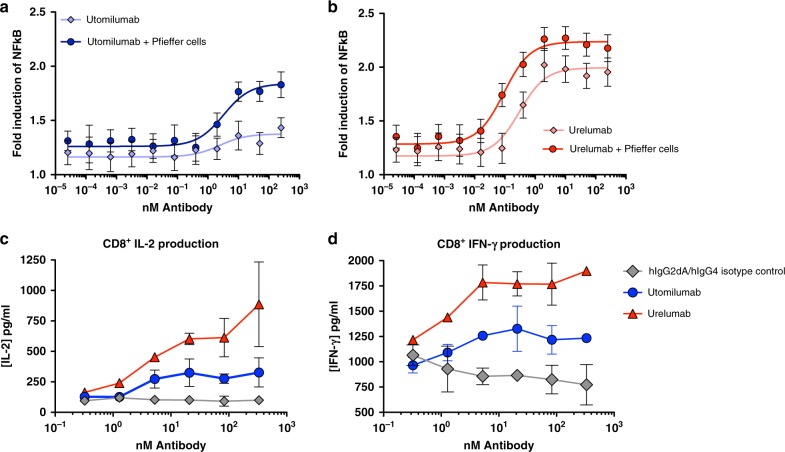
Urelumab activates 4-1BB signaling more strongly than utomilumab. Human 4-1BB NF-ĸB luciferase reporter HEK293 cells were incubated with fivefold increasing concentrations (max 250 nM) of **a** utomilumab or **b** urelumab antibody at 37 °C for 6 h before measuring luciferase activity and comparing to unstimulated cells. Mean fold induction was plotted against concentrations of test antibodies. Error bars represent the propagated standard deviation (SD) of three measurements. Solid lines are fits with dose-response curves with maximal fold induction of 1.38 ± 0.051 and 1.84 ± 0.053 for utomilumab and 1.99 ± 0.046 and 2.24 ± 0.033. **c**, **d** Purified human CD8 T cells were stimulated with plate bound anti-CD3 (50 ng/ml) plus each plate bound antibody with fourfold increasing concentrations (max 333.33 nM) at 37° C for 72 h and human cytokine production was measured by ELISA. Error bars represent the standard deviation of two measurements. All cytokines are measured from the same donor

In addition to our structural characterization, anecdotal reports of differences in activity between utomilumab and urelumab led us to examine their activity in cell-based assays^21,23^. Agonist activities of the two antibodies were assessed using NF-ĸB luciferase reporter assay in a h4-1BB-expressing HEK293 cell line in the presence and absence of FcγRIIB cross-linking by B cells (Pfeiffer cells) to assess baseline agonism as well as the potential for increased agonism when antibody-receptor complexes are further cross-linked^50^. In the absence of cross-linking, utomilumab shows moderate agonistic activity with a 1.36-fold induction over unstimulated reporter cells at the highest concentration measured. The activity increases to 1.84-fold induction over unstimulated cells when cross-linked by Pfeiffer cells (Fig. 7a). Urelumab shows stronger agonist activity in the absence of cross-linking with 1.99-fold induction over unstimulated cells. This activity is further increased to 2.24-fold induction in the presence of Pfeiffer cells (Fig. 7b). When we compared activity in a h4-1BB-expressing Jurkat cell line, similar trends were seen with urelumab showing much more pronounced activity. Negative control hIgG2 and hIgG4 antibodies showed no activation (Supplementary Figure 10AB, C). To further compare the activities of both antibodies, utomilumab and urelumab were evaluated in isolated human primary CD8+ T cells for their ability to stimulate anti-CD3-mediated cytokine secretion. Consistent with the results from 4-1BB expressing HEK293 cells, utomilumab stimulates intermediate production of IFN-γ and IL-2 while urelumab drives a stronger cytokine response (Fig. 7c, d).

**Fig. 8:**
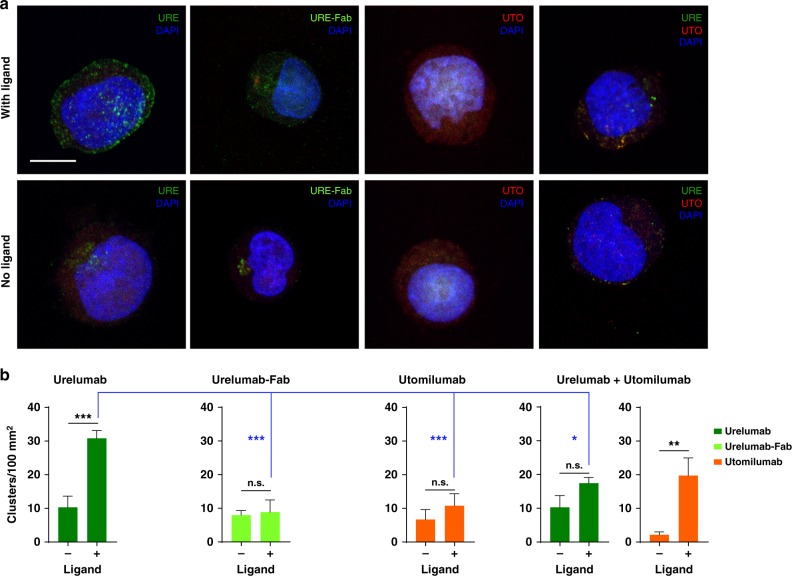
Differential clustering of 4-1BB receptors on Jurkat cells induced by urelumab and utomilumab. **a** Confocal images of 4-1BB receptor co-clusters with Urelumab-Alexa Fluor 488 conjugate (URE, green), Urelumab-Fab Alexa Fluor 488 Conjugate (URE-Fab), Utomilumab Alexa Fluor 594 conjugate (UTO, red), or with a combination of URE and UTO in the presence (top row) or absence (bottom row) of 4-1BB His-Avi ligand. Nuclei are stained with DAPI (blue). Scale bar = 10 µm. **b** Numbers of observed clusters per 100 µm^2^ of cellular area. Data shown as mean + S.E.M., *N* = at least 10 cells from three independent experiments. *p-value ≤ 0.05; **p-value ≤ 0.01; ***p-value ≤ 0.001; n.s., not significant

Given that receptor clustering is a prerequisite for downstream signaling, we tested whether urelumab or utomilumab could potentiate differential clustering of 4-1BB on cells in the presence or absence of 4-1BBL. We hypothesized that urelumab should induce strong ligand-dependent clustering as our structure suggests that the antibody can freely cross-link receptors trimerized by the ligand. Conversely, utomilumab would show reduced clustering. Confocal imaging showed significantly increased clustering of 4-1BB receptor above basal levels with urelumab IgG that was ligand dependent (Fig. 8a). As expected urelumab Fab was unable to induce clustering, consistent with our model. Additionally, utomilumab failed to induce clustering, as anticipated from our structures, and, finally, urelumab-induced clustering of 4-1BB receptors was significantly reduced by a combination of urelumab and utomilumab consistent with utomilumab’s ability to block ligand binding (Fig. 8b).

## Discussion

As an initial step to understanding the differences in how utomilumab and urelumab perturb 4-1BB signaling, we solved the structure of the 4-1BB/4-1BBL complex showing that it shares the same overall architecture of many of the TNF receptor–ligand complexes. The three copies of 4-1BB in the complex are arranged along parallel axes around the 4-1BBL trimer in what is presumably the activated state of the complex, allowing signaling through interactions of the clustered cytoplasmic tails of the receptor with TRAF1/2^51,52^. Notably, our structure reveals a non-canonical binding modality among the TNF family members where the receptor interaction is dominated by a single ligand in the trimer with very little interaction with the adjacent ligand protomer. Additionally, with the 4-1BB/4-1BBL structure we confirmed the previously hypothesized domain organization of the receptor, and we determined that our human 4-1BB receptor structure is very similar to the recently reported murine 4-1BB, though with potential differences in glycosylation^35,46,47^. Furthermore, with the additional antibody-bound structures of the receptor we now have a new appreciation for the range of motion of 4-1BB seen at the pivot point between the A2 and A1 motifs of CRD-3. Interestingly, this hinge point sits in the middle of the utomilumab binding site which would force 4-1BB to adopt a bent conformation and may affect receptor signaling activity by restricting its movement (Fig. 6). However, it remains to be seen what role receptor flexibility plays in the ligand engagement or downstream signaling or, indeed, if this is a feature shared across other TNFRSF members.

One of the noted features of our 4-1BB/4-1BBL complex was that the arrangement of the ligand trimer in the complex diverged from the previously published 4-1BBL structure. We solved the structure of 4-1BBL trimer alone to verify if the trimer transitions to the pinwheel-like arrangement in the absence of receptor as seen in PDB 2X29. However, in our structures, we found that the arrangement of our ligand trimer alone and ligand trimer bound to receptor are almost identical (Supplementary Figure 7). Interestingly, comparisons between our 4-1BBL structure and the previously solved ligand structure show that the overall fold of the individual ligand protomers are largely the same with protomers between structures superimposing with an RMSD of 0.76 Å^2^ (Supplementary Figure 4C). One exception is in the N-terminus of the previous ligand structure where residues 80–88 insert into the outer beta sheet, displacing the B′ strand. This particular strand arrangement deviates from the B′BGDE topology seen in other TNFSF structures. However, while the previous ligand structure shows an altogether novel arrangement for a TNF ligand trimer, our 4-1BBL structure shows the canonical bell-shaped trimeric assembly and largely conserved inter-subunit interfaces. One of the key differences between these two 4-1BBL structures is that the previous extended pinwheel structure was purified in the presence of non-ionic detergents NP-40 (0.1%) and dodecyl octaethylene glycol ether (0.1%), both of which have the capacity to interact with exposed hydrophobic residues. In fact, close examination of electron density maps from PDB code 2X29 show the presence of strong unmodeled 2Fo-Fc and positive difference density (1.5*σ* and 3*σ*, respectively) near the hydrophobic interface of the protomers that could easily accommodate the aliphatic tail of a detergent molecule and account for the unusual pinwheel-like conformation of the 4-1BBL trimer (Supplementary Figure 4D). Hence, we believe our 4-1BBL structure represents the native unbound structure given its agreement with 4-1BB/4-1BBL complex as well as other structures of the TNFSF members either alone or in complex with their respective receptors.

Both utomilumab and urelumab bind 4-1BB with similarly high affinity. Our SPR experiments measuring monovalent binding of utomilumab and urelumab to wild type h4-1BB give *K*_D_s of 69 and 22 nM, respectively (Supplementary Figure 8). Despite their similar binding affinities these antibodies demonstrate distinct differences in their agonist activity and reported toxicity. These data suggest that the determinants contributing to differences in activity and toxicity are likely more complex than simple binding kinetics. In fact, the antibodies bind 4-1BB in markedly different modes. Utomilumab binds in the center of 4-1BB at the junction of CRDs 3 and 4 while urelumab binds distally to utomilumab on the N-terminus of CRD1. Combined with the structure of 4-1BB/4-1BBL complex, we can now appreciate the impact these antibodies could have on receptor cross-linking, receptor–ligand interactions, how the antibodies are oriented relative to the cell surface and, ultimately, their impact on activation of the 4-1BB signaling axis. The mechanism for urelumab toxicity in patients is still an area of active research. While the two antibody-bound structures of 4-1BB presented here do not definitively explain differences in toxicity or activity, they raise several possibilities. First, both IgG2 and IgG4 are known for limited FcγR engagement and reduced antibody dependent cell-mediated toxicity (ADCC)^20,53,54^. The urelumab binding site on 4-1BB orients the antibody such that the Fc domain would be optimally exposed for interaction with FcγR, potentially enhancing what limited ADCC that could occur. Conversely, utomilumab binds closer to the cell surface and is oriented parallel to the membrane where engagement of FcγR may be more restricted. Furthermore, in murine models of antibody-driven 4-1BB agonism, observed toxicity was not dependent on either stimulatory FcγRs or the complement system^55^. Hence, it is still unclear what role FcγR engagement may play in human patients. Additionally, epitope accessibility may play role in toxicity and agonist activity of these antibodies. Again, urelumab’s binding site is on the very N-terminus of 4-1BB at CRD-1 where it is likely maximally exposed on the surface of the cell while utomilumab’s binding site at CRDs 3 and 4 may be sequestered or obscured by other surface molecules. However, both antibodies showed similar qualitative increases in our cell-based activity assays when cross-linked through FcγRIIB by Pfeiffer cells.

There is a clear difference between antibodies in ligand-blocking ability. Importantly, the utomilumab binding site lies on the same face as the ligand binding site. While the ligand and utomilumab binding sites do not directly overlap, they are adjacent. Rather than directly blocking the binding site of the ligand, utomilumab sterically occludes the ligand interaction which is clearly evident when the receptor-bound structure of utomilumab is overlaid over the structure of the receptor–ligand complex (Fig. 6a). In the overlapping structure, utomilumab’s major steric clash is with the adjacent ligand monomer which is only minimally engaged with the antibody-bound receptor. Indeed, in our sandwich assay, utomilumab prevents 4-1BBL binding to receptor (Fig. 5a). Given its higher affinity, it would outcompete ligand for receptor occupancy, as would be expected for a classical ligand blocker. Unlike utomilumab, urelumab binding to 4-1BB does not appear to interfere with receptor engagement of the ligand. Instead urelumab binds independently of 4-1BBL with the bulk of the Fab facing away from the center axis of the receptor–ligand complex (Fig. 6b). Furthermore, our sandwich assay demonstrates that urelumab is capable of forming a ternary complex with receptor and ligand (Fig. 5b). Cross-linking 4-1BB through the bivalent binding of the IgG, the orientation of urelumab on CRD-1 allows for the possibility of cross-linking multiple 4-1BB/4-1BBL complexes, further augmenting its agonist properties. While the ability for IgGs to permeate the immunological synapse is not well-understood, such interactions could additionally occur in the presence of proteolytically shed 4-1BB as seen in patients with hematological malignancies such as myelodysplastic syndrome and acute myeloid leukemia^56,57^. Conversely, each utomilumab IgG is only capable of cross-linking two receptors, limiting the antibody to more traditional modes of agonism.

When we looked at activation of 4-1BB signaling using our 4-1BB-transduced HEK293 NF-ĸB luciferase cell line, we found that in solution both antibodies are able to activate 4-1BB signaling either alone and, to a greater extent, with Pfieffer cell cross-linking. This is in contrast with previous utomilumab assay data that required cross-linking or plate bound antibody to observe activity^18^. Utomilumab demonstrated more moderate activation and required higher concentrations to reach its maximum activation. We were able to recapitulate these results in the more complex setting of CD3-stimulated purified human CD8^+^ T cells where we saw greater IL-2 and IFN-γ cytokine induction by urelumab than utomilumab at comparable antibody concentrations. This demonstrates that the epitopes, separate from ligand-dependent clustering or ligand blocking, are affecting activation of 4-1BB, potentially driven by epitope accessibility, as noted above. The increased activity seen in the presence of FcγRIIB mediated cross-linking by Pfeiffer cells demonstrates that both antibodies have the capability for increased agonism in the presences of this B cell line, and suggests that urelumab epitope geometry might allow greater activation than utomilumab overall, similar to the complex interplay between epitope specificity and biological activity seen with CD40 antibodies^58^.

Previous examples of enhanced antibody agonism in the presence of TNF ligand has been shown in the case of the death receptor 5 targeting antibody AMG655 and the Apo2L/TRAIL ligand where the antibody epitope permitted concomitant binding of both antibody and ligand allowing for higher order clustering^59^. Here, using confocal microscopy on our 4-1BB-transduced Jurkat cell line we demonstrated that urelumab can, indeed, drive ligand-induced clustering on cells while utomilumab does not, and that utomilumab can moderate urelumab’s clustering ability presumably by blocking binding of 4-1BB receptor to its ligand and limiting the formation of higher order structures. Bivalent binding of urelumab is required, not just interaction with CRD-1, as the urelumab Fab does not cluster receptor–ligand complex. The ability of urelumab to induce clustering and utomilumab’s ligand blocking could explain how the binding epitopes for each of the antibodies can, at least in part, contribute to the distinct agonist activity profiles demonstrated by both antibodies.

The 4-1BB signaling axis is currently an area of intense interest for the development of immuno-oncology therapeutics. The lack of structural data for both 4-1BB and its complex with 4-1BBL have made it difficult to fully evaluate antibodies targeting this pathway. The structures we present of the 4-1BB/4-1BBL complex and the 4-1BBL trimer alone help fill that knowledge gap as well as provide evidence that 4-1BBL adopts the canonical trimeric arrangement shared among the TNFSF members in contrast with previous data. With the structures of utomilumab and urelumab bound to 4-1BB and accompanying activity and clustering data, we establish clear differences in antibody epitopes and provide insight into how antibodies binding these epitopes could affect 4-1BB signaling. Taken together, these data provide a structural road map toward the development of new therapeutics that may have more tailored effects on 4-1BB activity.

## Methods

### Protein expression and purification

Proteins used for crystallization were expressed and purified as follows. DNA sequences for human 4-1BBL (ReqSeq NP_003802) residues 58–254 and human 4-1BB (RefSeq NP_001552) residues 25–162 were synthesized with an N-terminal secretion sequence and a C-terminal 8x His tag (ligand) or TEV cleavage sequence followed by an 8x His tag (receptor) and cloned into the pFastBac vector with EcoRI/HindIII and expressed, following the protocols from the Bac-to-Bac Baculovirus Expression System (Gibco, #10359016) in HighFive insect cells (Gibco, #B85502) for 48 h. Media containing the secreted protein was harvested by centrifugation and filtration before purification by immobilized metal affinity chromatography on a Ni Sepharose excel column (GE) equilibrated in 25 mM Tris pH 8.0, 500 mM NaCl. Bound protein was washed with 25 mM Tris pH 8.0, 500 mM NaCl, 15 mM imidazole before eluting with 25 mM Tris pH 8.0, 500 mM NaCl, 250 mM imidazole. Protein was further purified by size exclusion chromatography over a Superdex 200 column (GE) equilibrated in 10 mM HEPES pH 7.5, 150 mM NaCl. Additionally, 4-1BB receptor was digested overnight with TEV protease (Accelagen) between Ni Sepharose and Superdex purification steps for the removal of the His-tag.

Sequences corresponding to the utomilumab lambda light chain and human IgG1 heavy chain Fab region (Supplementary Table 2) with a C-terminal 10x His tag were synthesized with an N-terminal secretion signal and inserted into an in-house mammalian expression vector with restriction enzymes and co-transfected into HEK cells (Gibco, # A14528) for expression. After 72 h, media containing secreted Fab was harvested by centrifugation and filtration before purification by on a Ni Sepharose excel column (GE) equilibrated in 25 mM Tris pH 8.0, 500 mM NaCl. Bound protein was washed with 25 mM Tris pH 8.0, 500 mM NaCl, 15 mM imidazole before eluting with 25 mM Tris pH 8.0, 500 mM NaCl, 250 mM imidazole. Protein was further purified by size exclusion chromatography over a Superdex 75 column (GE) equilibrated in 1xPBS (Corning). Urelumab Fab was purified following the same protocol.

4-1BB/4-1BBL complex and 4-1BB receptor and urelumab or utomilumab Fab complexes were made by mixing receptor and ligand or receptor and Fab at a 1.5:1 molar ratio and incubating on ice for 1 h before further purification by size exclusion chromatography over a Superdex 200 column (GE) equilibrated in 10 mM HEPES pH 7.5, 150 mM NaCl.

For ELISA, NF-ĸB activation and cytokine release assays, utomilumab (fully human IgG2dA mAb), urelumab (fully human IgG4 mAb), and the anti-bovine herpesvirus (BHV) hIgG2 and hIgG4 isotype controls were expressed in HEK cells. Media containing secreted IgG was harvested by filtration and purified over Protein A (MabSelect SuRe, GE) before dialysis into 1xPBS (Corning).

For surface plasmon resonance experiments, ELISA and confocal microscopy experiments, human 4-1BBL residues 58–254 (wild type and R171A mutant) and human 4-1BB residues 24–186 (wild type, N42R, I64R, V71R, M101R, and I132R) were subcloned into mammalian expression vectors with secretion sequences and a TEV cleavage site followed by a C-terminal 8x His–Avi tag and transfected into HEK cells for expression. After 72 h, media containing secreted 4-1BBL was harvested by centrifugation filtration before purification by immobilized metal affinity chromatography (NiExcel, GE) followed by size exclusion chromatography over a Superdex 200 column (GE) equilibrated in 1xPBS (Corning). When necessary, wild type and mutant 4-1BBL were subsequently biotinylated for surface capture using the BirA Biotin-protein ligase kit (Avidity) following the manufacture’s protocol.

### Protein crystallization

Purified 4-1BBL, 4-1BB/4-1BBL complex and 4-1BB–utomilumab and urelumab Fab complexes were screened for crystallization hits in sitting drop 96-well format using a mosquito liquid handling robot (TTP Labtech). Hits were translated to hanging-drop vapor diffusion and crystals that were suitable for diffraction experiments were harvested, cryoprotected, flash cooled, and stored in liquid nitrogen for transport to the beamline.

4-1BBL was crystallized at 6.9 mg/ml in 8% w/v PEG 8000, 100 mM Tris 8.5. Crystals were cryoprotected in the same condition with the addition of 25% v/v glycerol. 4-1BBL crystals diffracted to a maximum resolution of 2.9 Å in space group P62. 4-1BB/4-1BBL complex was crystallized at 11.5 mg/ml in 2.5 M sodium acetate pH 7.0 plus 0.1% w/v ovalbumin, 0.0005% w/v pepsin, 0.0005% w/v proteinase K, 0.0005% w/v trypsin, 0.002 M HEPES sodium pH 6.8. Crystals were cryprotected in 3 M sodium acetate pH 7.0 plus 25% v/v glycerol. 4-1BB receptor–ligand complex crystals diffracted to a maximum resolution of 2.1 Å in space group C2221. 4-1BB–utomilumab Fab complex was crystallized at 15.9 mg/ml in 1.5 M lithium sulfate, 0.1 M HEPES pH 7.0. Crystals were cryoprotected in 1.6 M lithium sulfate plus 20% v/v glycerol. 4-1BB receptor–utomilumab Fab complex crystals diffracted to a maximum resolution of 2.7 Å in space group I2. 4-1BB–urelumab Fab complex was crystallized at 8 mg/ml in 1 M sodium malonate, pH 7.0, 0.4% v/v Jeffamine ED-2001 pH7.0, 0.1 M HEPES pH7.0. Crystals were cryoprotected in the same condition plus 25% v/v glycerol. 4-1BB receptor–urelumab Fab complex crystals diffracted to a maximum resolution of 2.8 Å in space group C2221.

### Data collection and structure determination

Diffraction images were collected at the Advanced Light Source beamlines 5.0.1 and 5.0.2 on a Pilatus detector (Dectris) and were indexed, integrated and scaled using XDS^60^. All diffraction experiments were carried out 100K. Data were collected at a wavelength of 0.977 Å for 4-1BBL, 4-1BB/4-1BBL complex and 4-1BB/utomilumab complex and 1.127 Å for 4-1BB/urelumab complex. Resolution limits were cut off at CC1/2 = 0.3^61^. Phases were determined by molecular replacement with Phaser^62^ using either the previous 4-1BBL structure (PDB code: 2X29) or a structure of a human IgG1 Fab with the CDR loops removed as search models. Structure refinement was carried out using PHENIX^63^ and structure validation performed using MolProbity^64^. Ramachandran statistics for 4-1BBL are 94.13% favored, 0.45% outliers. Ramachandran statistics for the 4-1BB/4-1BBL complex are 97.92% favored, 0.12% outliers. Ramachandran statistics for the 4-1BB/utomilumab complex are 95.54% favored, 0% outliers. Ramachandran statistics for the 4-1BB/urelumab complex are 94.43% favored, 0% outliers. Model inspection and manual rebuilding was performed using COOT^65^. Figures and structural alignments and superpositions were generated using PyMOL and UCSF Chimera^66,67^. Final data collection and refinement statistics are listed in Supplementary Table 1.

### Antibodies

Utomilumab (a fully human IgG2dA mAb), urelumab (a fully human IgG4 mAb), and the anti-bovine herpesvirus (BHV) hIgG2dA and hIgG4 isotype controls were biotinylated using EZ-Link Sulfo-NHS-LC-Biotin (Thermo Fisher Scientific) following manufacturer protocol and desalted using PD-10 Desalting Columns (GE Healthcare Life Sciences) following manufacturer protocol.

### ELISA

His-tagged 4-1BB WT or arginine mutants were captured at 4 °C overnight by a His Tag Antibody Clone # AD1.1.10 (R&D Systems). 10 µg/ml of utomilumab, urelumab, or biotinylated 4-1BBL were then captured at RT for 1 h and binding was detected by the addition of anti-human IgG Fc-HRP (Jackson ImmunoResearch) or streptavidin-HRP (R&D Systems) followed by ABTS Peroxidase Substrate (KPL). All experiments were done in triplicate.

### SPR analysis of 4-1BB binding to 4-1BBL

The affinities for the interactions of human 4-1BBL wild type and R171A mutant with human 4-1BB were determined on a Bio-Rad ProteOn XPR36 SPR instrument (Bio-Rad Laboratories, Hercules, CA) equipped with a Bio-Rad NLC (neutravidin-coated) sensor chip (catalog No. 1765021, Bio-Rad Laboratories, Hercules, CA). Surfaces were prepared in HBS (10 mM HEPES, 150 mM NaCl, pH 7.4) supplemented with 0.01% (v/v) Tween-20 as running buffer at 25 °C. Biotinylated human 4-1BBL R171A mutant was diluted to 1 µg/mL into running buffer and captured along one vertical channel of the NLC sensor chip at a flow rate of 30 µL/min for 150 s. Biotinylated human 4-1BBL wild type was also diluted to 1 µg/mL into running buffer and captured along a second vertical channel at a flow rate of 30 µL/min for 300 s. Buffer was injected along the remaining vertical channels. The final immobilization level for biotinylated human 4-1BBL R171A mutant was 516 ± 12 RU and for biotinylated human 4-1BBL wild type was 543 ± 21 RU.

Kinetic assays were conducted at 37 °C with HBS supplemented with 0.01% (v/v) Tween-20 and 1 mg/mL of bovine serum albumin (BSA) as running buffer. A one-shot kinetic method was implemented^68^. Human 4-1BB at concentrations of 3.2, 16, 80, 400, and 2000 nM, and buffer were injected as analytes at a flow rate of 30 µL/min for 120 s along the horizontal channels of the sensor chip and dissociation was monitored for 600 s. Each interaction was measured in triplicate using three independent analyte dilution series. Human 4-1BB was allowed to dissociate to baseline before measuring replicates.

Data were referenced using the interspot reference and then double-referenced with the data from the buffer analyte injections^69^. Data were fitted by steady-state analysis using ProteOn Manager Software (version 3.1.0).

### Utomilumab or urelumab blocking of 4-1BB/4-1BBL interactions

Blocking of the interaction of human 4-1BB with human 4-1BBL was performed in a classical sandwich assay format on a Biacore T200 SPR instrument (GE Lifesciences, Marlborough, MA) at 25 °C.

An anti-human kappa/anti-human lambda capture chip was prepared by amine-coupling goat F(ab′)_2_ anti-human kappa (catalog No. 2063-01, SouthernBiotech, Birmingham, AL) and goat anti-human lambda (catalog No. 2071-01, SouthernBiotech, Birmingham, AL) to a Biacore Series S sensor chip CM4 (catalog No. BR100534, GE Lifesciences, Marlborough, MA) surface at 25 °C. The running buffer for the immobilization procedure was HBS supplemented with 0.05% (v/v) Tween-20. The anti-human kappa was diluted to 50 µg/mL into 10 mM sodium acetate pH 4.5, and injected in flow cells 1 and 2 at 20 µL/min for 7 min after activation of flow cell 1 and 2 surfaces with a 1:1 (v/v) mixture of 400 mM 1-Ethyl-3-(3-Dimethylaminopropyl) carbodiimide hydrochloride (EDC) and 100 mM N-hydroxysuccinimide (NHS) for 7 min at 10 µL/min. Excess reactive esters on the surface of flow cells 1 and 2 were blocked for 7 min at 10 µL/min with 100 mM ethylenediamine in 200 mM borate buffer pH 8.5. The anti-human lambda was also diluted to 50 µg/mL into 10 mM sodium acetate pH 4.5, and injected in flow cells 3 and 4 at 20 µL/min for 7 min after activation of the surfaces in flow cells 3 and 4 with a 1:1 (v/v) mixture of 400 mM EDC and 100 mM NHS for 7 min at 10 µL/min. Excess reactive esters on the surface of flow cells 3 and 4 were blocked for 7 min at 10 µL/min with 100 mM ethylenediamine in 200 mM borate buffer pH 8.5. Surfaces in flow cells 1 and 2 were conditioned with three, 30-s injections of 10 mM glycine-HCl pH 1.7 at 10 µL/min, while surfaces in flow cells 3 and 4 with three, 30-s injections of 10 mM glycine-HCl pH 2.0.

The running and dilution buffer for the classical sandwich assay was HBS supplemented with 0.05% (v/v) Tween-20 and 1 mg/mL BSA. Urelumab Fab at 10 µg/mL was captured onto the anti-human kappa surface on flow cell 2 for 2 min at 10 µL/min. Similarly, utomilumab at 10 µg/mL was captured onto the anti-human lambda surface on flow cell 4 for 2 min at 10 µL/min. Buffer or human 4-1BB at 100 nM as the “First Analyte” was then injected in all flow cells for 2 min followed by a 10-s injection of buffer at a flow rate of 10 µL/min. Then, buffer, human 4-1BBL (catalog no. 2295-4L/CF, R&D Systems, Minneapolis, MN) at 100 nM, or anti-Avi (catalog no. A00674, Genscript, Piscataway, NJ) at 100 nM as the “Second Analyte” was injected for 2 min at 10 µL/min in all flow cells and dissociation was monitored for 3 min. The anti-human kappa surfaces were regenerated using 330-s injections of 10 mM glycine-HCl pH 1.7 at 10 µL/min while the anti-human lambda surfaces were regenerated using 330-s injections of 10 mM glycine-HCl pH 2.0 at 10 µl/min. Flow cells 1 and 3 were used as references for flow cells 2 and 4, respectively. The experiment was run in triplicate using 3 independent dilutions for each sample.

The anti-Avi was used to show that human 4-1BB is present and to demonstrate sandwiching to human 4-1BB when human 4-1BB is captured by either utomilumab Fab or urelumab Fab since human 4-1BB has an Avi-tag.

### Cell culture

HEK293 cells were passaged in DMEM (Corning; #10-013-CV) with 10% FBS (Hyclone; #SH30070.03), 2 mM Gluta-gro (Corning; #25-015-Cl), and 20 ml/L penicillin:streptomycin solution (Corning 30-002-Cl). Jurkat cells were passaged in RPMI 1640 (Corning; #10-041-CV) with 10% FBS, 2 mM Gluta-gro, and 20 ml/L penicillin:streptomycin solution. Cells were grown at 37 C^o^/5% CO_2_.

### Cell line generation

To assay 41BB signaling in HEK293 cells, a 41BB expression cassette and a luciferase reporter were integrated at a single genomic locus using the Flp-In System (Thermo Fisher Scientific). DNA encoding a NFkB response element, a minimal promoter, a luciferase, and a SV40 polyadenylation sequence was inserted into pEF5/FRT (Thermo Fisher Scientific; #K601002) through restriction cloning with *NsiI* and *SpeI*. A cassette encoding the CMV promoter followed by human 41BB (Uniprot ID: Q07011-1) was then inserted using *SpeI* and *NotI*. The resulting plasmid were then transfected into HEK293 Flp-In cells (Thermo Fisher Scientific; #R75007) along with a plasmid encoding Flp Recombinase (pOG44) using Lipofectamine 200 (Invitrogen; #11668500), and integrated cells were selected by resistance to 75 μg/ml Hygromycin (Gibco; #10-687-010).

In order to investigate 41BB signaling in a lymphoid cell line, a Jurkat NFkB Reporter cell line (BPSBioscience, #60651) was transduced with a 41BB expression cassette. For activity assays, TurboGFP-P2A-41BB was cloned into a 2nd generation lentivirus plasmid (pLVX-EF1α by Clontech; #631253). For microscopy studies, the EF1α promoter was replaced by 250 nucleotides from the PGK promoter (e.g., PGK250), and the β and γ components of the WPRE were deleted.

### Luciferase assays

20e3 HEK293 reporter cells were plated on poly-L-lysine (Sigma; #P4707-50ML) coated 96-well plates (Corning; 07-200-587), or 50e3 Jurkat reporter cells on uncoated plates. Cells were incubated at 37 C^o^/5% CO_2_ in corresponding growth media, and agonists were added at the indicated concentrations either that day (for Jurkat cells) or the following day (for HEK293 cells). After 6 h, cells were lysed by incubation with One-Glo (Promega, #E6120) for 10 min, and luminescence was quantified by an Envision 2103 Multilabel Reader (Perkinelmer).

### In vitro human T cell cytokine release assay

Human T cells were isolated via negative selection from freshly isolated PBMC from human whole blood from a healthy volunteer donor using the Pan T Cell Isolation Kit (Miltenyi Biotec) following manufacturer protocol. Human CD8^+^ T cells were isolated via positive selection from freshly isolated T cells using CD8 MicroBeads (Miltenyi Biotec) following manufacturer protocol. One hundred thousand CD8^+^ T cells/well in 1x RPMI 1640/L-glutamine/25 mM HEPES (Corning), 10% FBS, 1x MEM nonessential amino acids, 10 mM HEPES, and 1x penicillin streptomycin were stimulated with 50 ng/ml of anti-CD3 clone UCHT-1 (eBioscience) plus fourfold increasing concentrations (max 50 µg/ml = 333.33 nM) of utomilumab, urelumab, or the isotype controls captured by anti-mouse and human IgG Fc antibodies (Jackson ImmunoResearch). 72 h post stimulation, human IFN-γ, TNF-α, and IL-2 production of the culture supernatants was measured by ELISA (R&D Systems).

### Confocal microscopy

Jurkat T cells expressing human 4-1BB receptor on PGK250 wpre-α were cultured using RPMI 1640 (Corning™) reconstituted with 10% FBS (JR Scientific), 100 U/mL penicillin (Gibco) 100 µg/mL streptomycin (Gibco), 2 mM L-glutamine (Gibco), 1 mM sodium pyruvate (Corning), 10 mM HEPES (Corning).

For staining Jurkat cells were freshly isolated from culture and washed with ice cold staining buffer (PBS + 2% FBS + 0.1% NaN_3_). 1E5 cells each were stained on ice with 2 µg/mL of (a) urelumab-Alexa Fluor 488 conjugate, (b) urelumab-Fab Alexa Fluor 488 conjugate, (c) utomilumab Alexa Fluor 594 conjugate, (d) urelumab Alexa Fluor 488+utomilumab Alexa Fluor 594 in staining buffer for 30 min in the presence or absence of 30 µg/mL of 4-1BBL-His Avi. Cells were then washed in staining buffer, fixed in 4% paraformaldehyde in PBS on ice for 10 min, permeabilized with 1x permeabilization buffer (Life Technologies), and then stained with 1:10,000 DAPI (Life Technologies) in staining buffer. Finally, cells were washed with staining buffer and mounted on cover glass chamber (iBidi™) in staining buffer. Cells were imaged using a Leica SPE confocal microscope equipped with a DMI4000 frame and a 100X HCX PL-APO 1.40 N.A. oil objective at a zoom setting of 3.0 and scan rate of 400 lines/s. Images were analyzed using Matlab (Mathworks Ltd.) by applying median noise reduction and local maxima for cluster detection. Clusters for each color were tallied separately. Clusters per 100 µm^2^ (average cellular area) have been shown. Statistical analyses were performed with Graphpad Prism utilizing one-way ANOVA with Tukey’s multiple comparison test. The Supplementary Information file contains a Supplementary Methods section with further details.

## Electronic supplementary material

Supplementary Information

## Data Availability

The data that support the findings of this study are available from the corresponding authors upon reasonable request. The atomic coordinates and structure factors have been deposit in the Protein Data Bank, www.pdb.org (PDB codes 6MGE, 6MGP, 6MI2, and 6MHR).
