# Supplementary material for: Structure of the 4-1BB/4-1BBL complex and distinct binding and functional properties of utomilumab and urelumab
Source: Nat Commun. 2018 Nov 8;9:4679. doi: 10.1038/s41467-018-07136-7 (PMC6224509; doi:10.1038/s41467-018-07136-7)
Supplement: Supplementary file 1 — Supplementary Information [file 41467_2018_7136_MOESM1_ESM.pdf]

Structure of the 4-1BB/4-1BBL complex and distinct binding and functional properties of utomilumab and urelumab

S.M. Chin, C.R. Kimberlin et al.

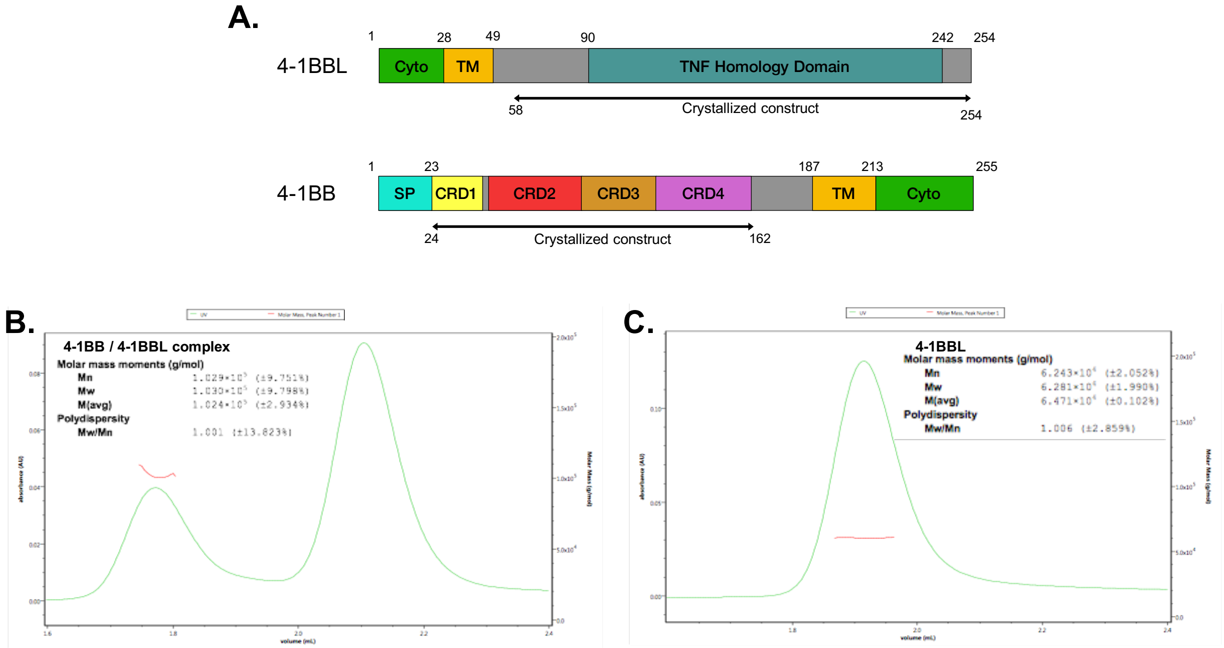

Supplementary Figure 1. Domain organization of 4-1BB and 4-1BBL and SEC-MALS analysis. **A**) Domain organization of the 4-1BB receptor and the 4-1BB ligand. Crystallized portions of each molecule are marked by black lines with inward facing arrows. Residue numbers at domain boundaries are indicated. Analysis of 4-1BB/ 4-1BBL complex (**B**) and 4-1BBL (**C**) by SEC-MALS gives an experimental molar masses of 103.0 kDa and 62.8kDa respectively, closely matching the calculated masses of 112kDa for the complex and 65.7 kDa for the ligand trimer.

A.

B.

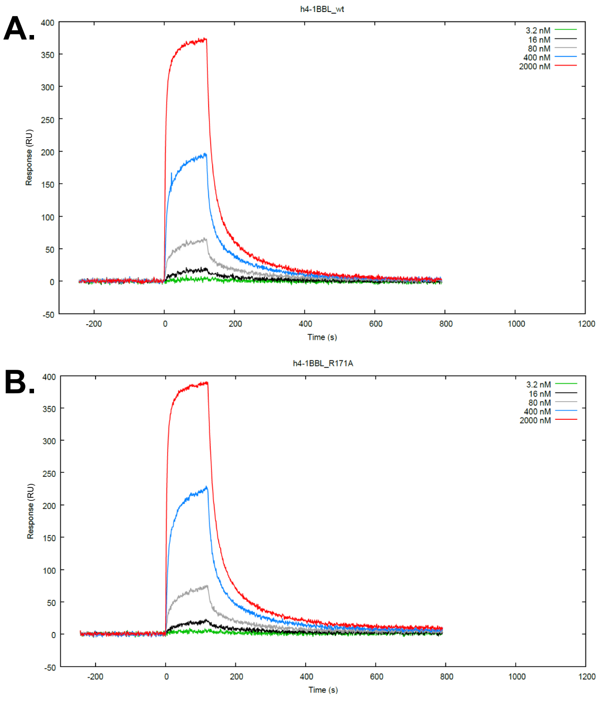

C.

Sample	T (°C)	N	K_D ±SD(nM)
4-1BBL R171A	37	3	521±17
4-1BBL WT	37	3	680±31

D.

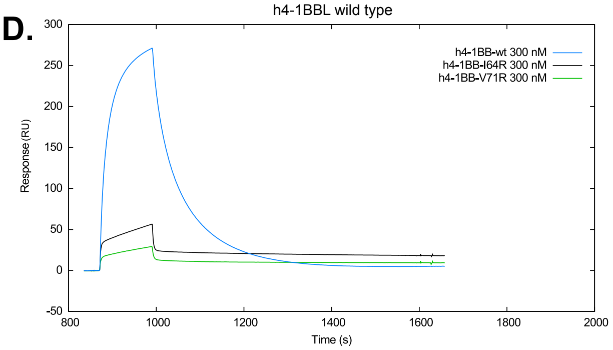

Supplementary Figure 2. Validation of h4-1BB/4-1BBL binding site. h4-1BB Binding to h4-1BBL and h4-1BBL R171A at 37°C. Interspot referenced and double-referenced sensorgrams from one replicate showing binding of h4-1BB receptor to (**A**) human 4-1BB ligand, and (**B**) human 4-1BB ligand with R171A mutation. Legend shows h4-1BB receptor concentrations used. Results from steady-state analysis of replicates summarized in (**C**). **D)** Double-referenced sensorgrams for h4-1BBL binding to h4-1BB mutants. Biotinylated h4-1BBL was captured with wild-type and mutant h4-1BB flowed as analyte at 300nM. Triplicate measurements are consistent with one another; therefore, one replicate is shown.

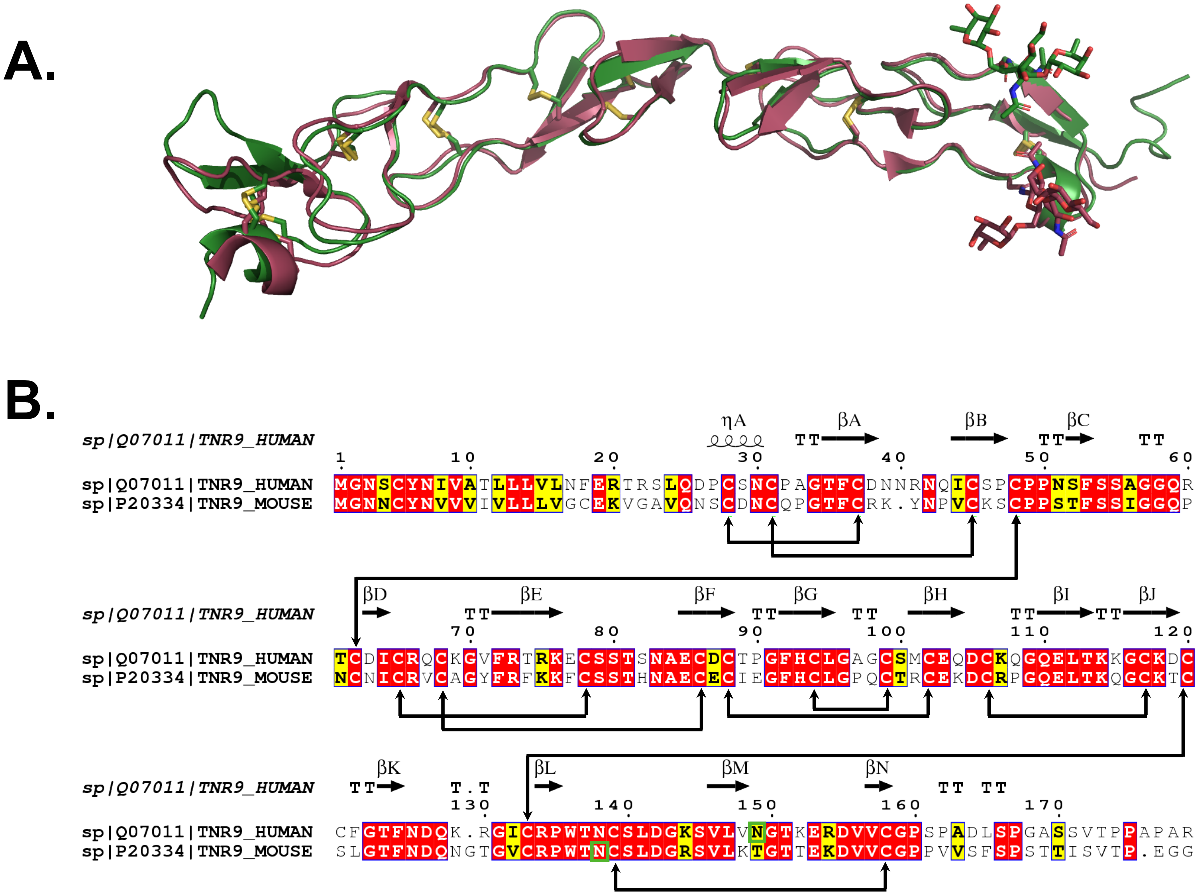

Supplementary Figure 3. Structural and sequence alignment of mouse and human 4-1BB receptor. **A**) Human (forest green) and mouse (raspberry, PDB code 5wi8) were aligned using the PyMOL superposition tool (RMS 0.7Å2, matching on 687 atoms). Protein backbone is shown in cartoon representation with disulfide bonds and glycosylation sites highlighted in sticks. Mouse 4-1BB receptor is glycosylated at N138 while human 4-1BB receptor is glycosylated at N149. **B**) Structure based sequence alignment of human and mouse 4-1BB receptor with human 4-1BB secondary structure elements and disulfide bonds highlighted (β-strands shown as arrows, *α*-helices shown as coils, β-turns shows as T). Identical sequence is highlighted in red, similar in yellow. Respective glycosylation sites are highlighted with green boxes.

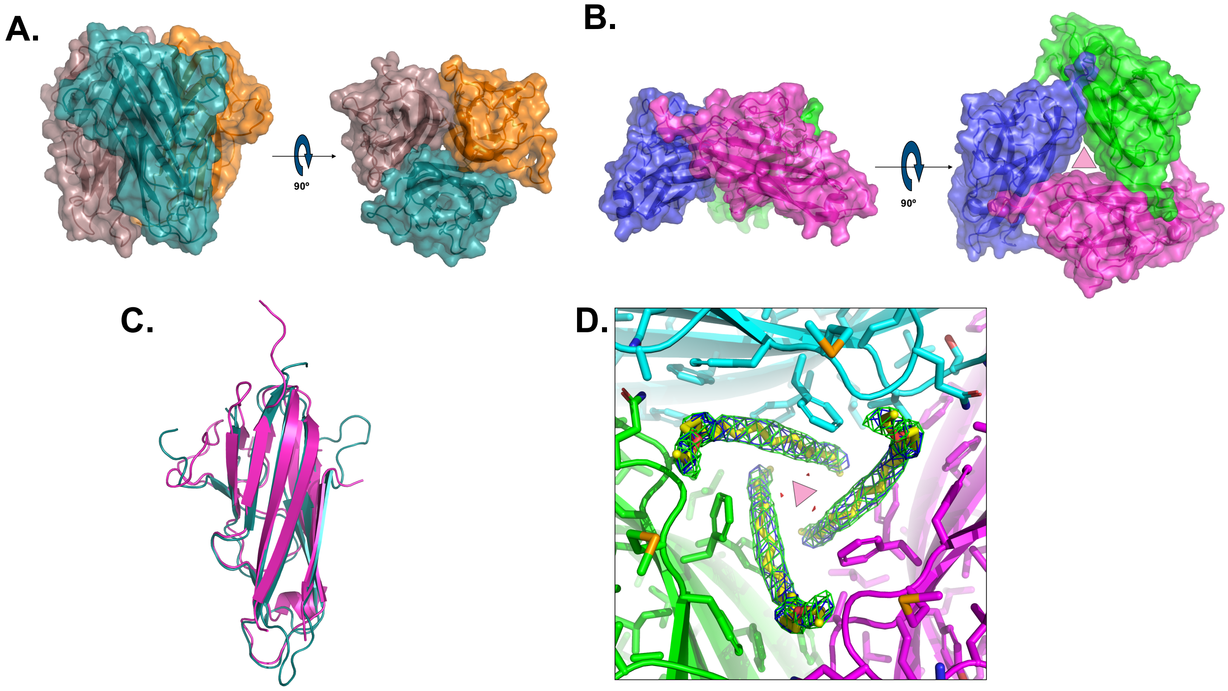

Supplementary Figure 4. Comparison with the previously solved 4-1BB ligand structure. Side and top down views of 4-1BB ligand structure, presented here in this paper (**A**) and solved previously (**B**)(PDB code 2X29). **C**) 4-1BBL protomers from 2X29 (violet) and our 4-1BBL structure (deap teal) align with an RMS of 0.76Å2 (matching on 623 atoms) **D**) Unmodeled density present in PDB code 2X29 maps near the 3-fold crystallographic symmetry axis (pink triangle). The 2Fo-Fc density (1.5σ, blue mesh) and Fo-Fc density (+/- 3.0σ, green and red mesh) easily accommodate the aliphatic tale of a doecyl octaethylene glycol ether detergent molecule (fit in density, carbon in yellow, oxygen in red).

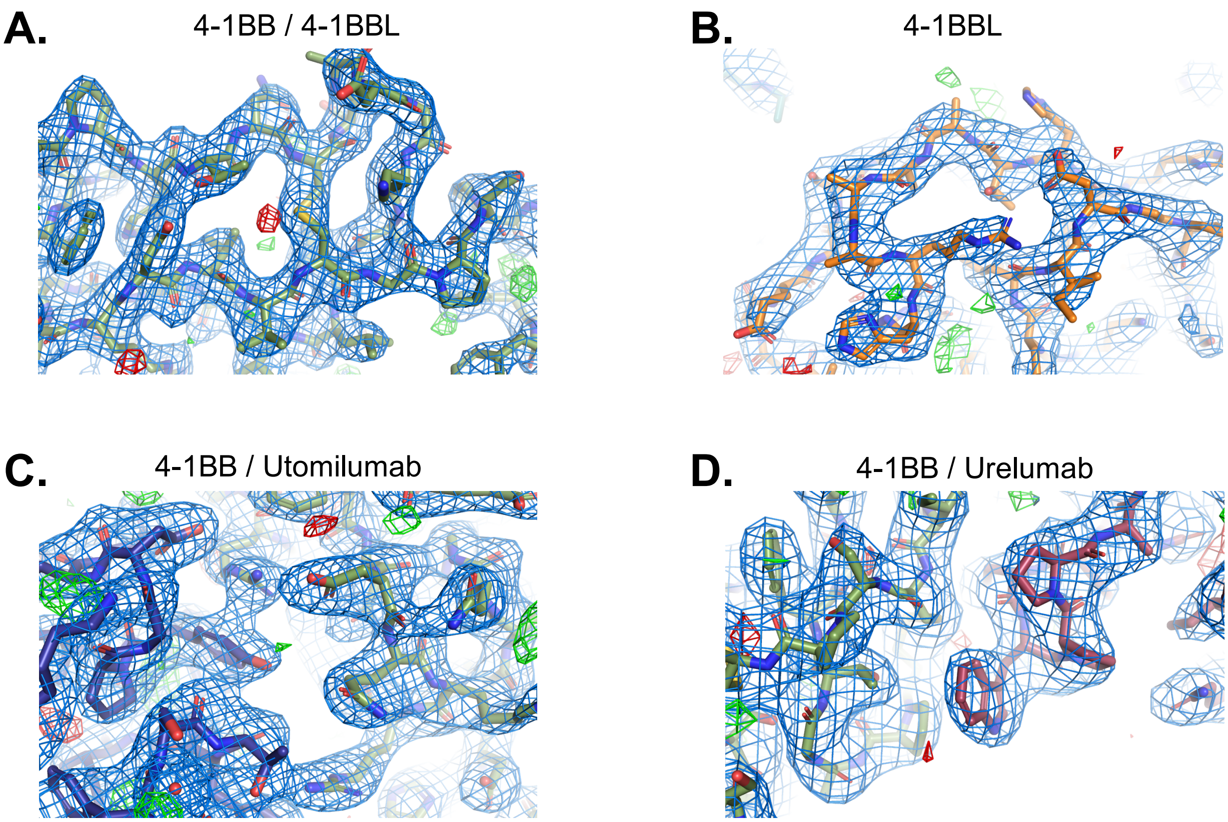

Supplementary Figure 5. Exemplar electron density shown for the reported structures. (**A**) Receptor chain X (smudge green) from the 4-1BB / 4-1BBL complex structure. (**B**) Ligand protomer chain A (orange) from 4-1BBL structure. (**C**) The interface between receptor chain F (smudge green) and Utomilumab heavy chain F (deep blue) of the 4-1BB / Utomilumab complex structure. (**D**) The interface between receptor chain F (smudge green) and Urelumab light chain F (raspberry) of the 4-1BB / Urelumab complex structure. Density shown from weighted 2Fo-Fc (1.0σ, blue mesh) and weighted Fo-Fc (±3.0σ, green and red mesh) maps. Nitrogen and oxygen atoms are colored blue and red, respectively.

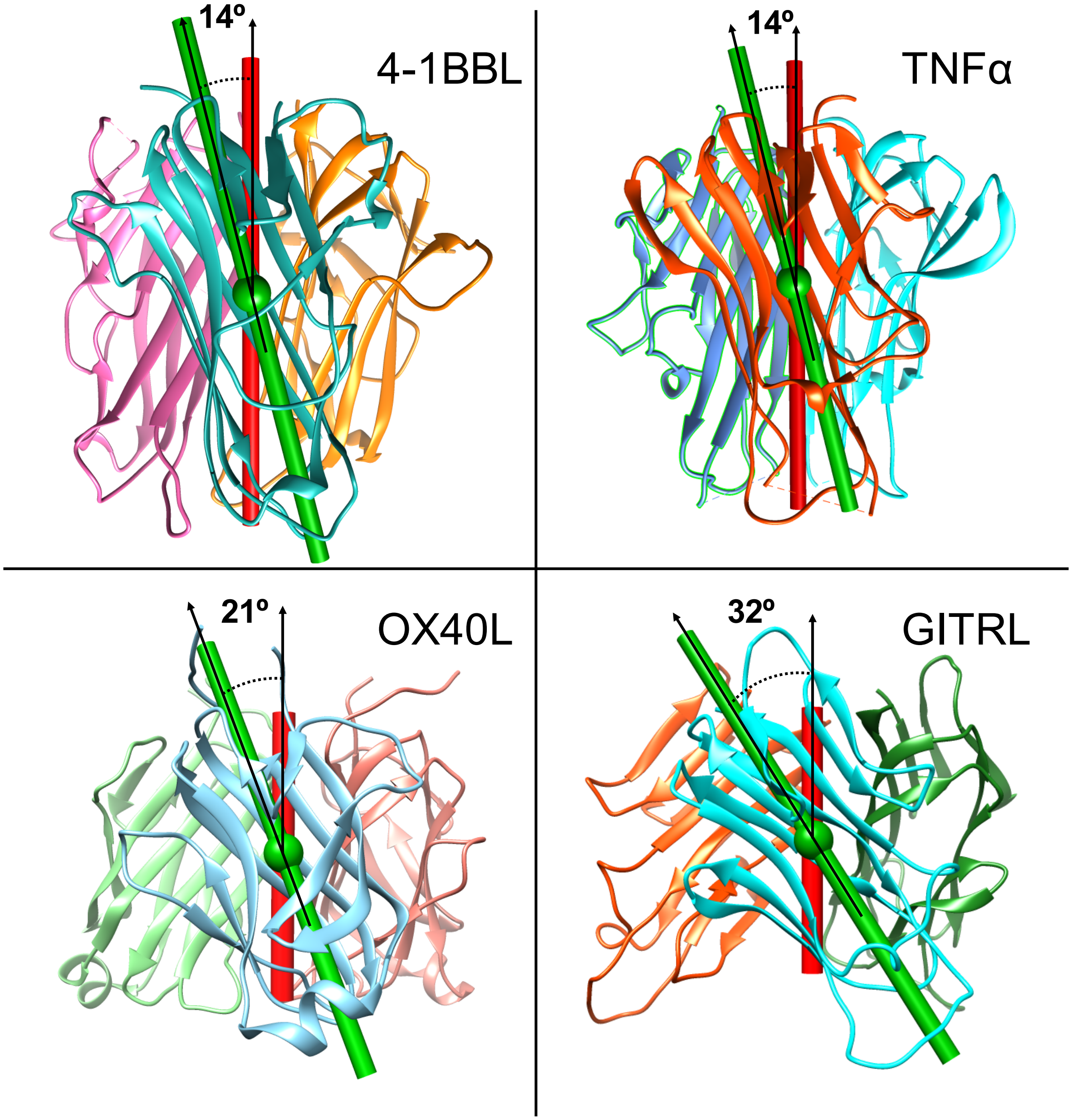

Supplementary Figure 6. Orientation of ligand protomers from different TNFSF members. The ligand trimer axes (red rods) and protomer centroid axes (green rods) were calculated for 4-1BBL, TNFα and divergent class members OX40L and GITRL using UCSF Chimera and the angle between axes measured. While other other members of the divergent group of TNFSF ligands show larger angles between axes and thus flatter trimeric assemblies, 4-1BBL is more similar to the conventional class of TNFSF ligands.

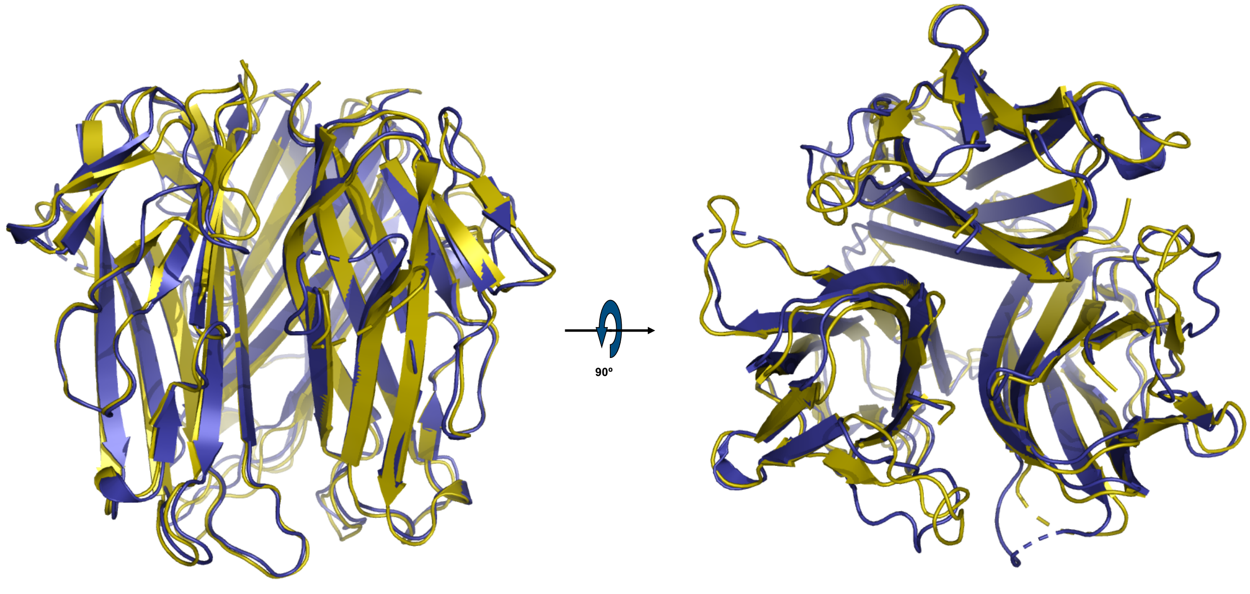

Supplementary Figure 7 Superposition of structures of 4-1BBL alone (olive) and 4-1BBL from the receptor - ligand complex (deep blue). Ligand protomers align with an overall RMSD of 0.778Å (matching on 2415 atoms).

A. **Sensorgrams for h4-1BB receptor (WT and mutants) binding to Utomilumab hIgG2 at 37°C**

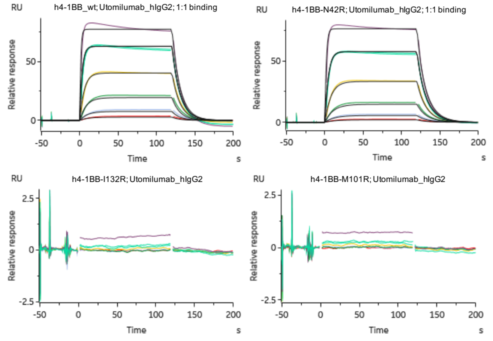

Binding kinetics and affinities of h4-1BB receptor (WT and mutants) to Utomilumab hIgG2 at 37°C

Sample	T (°C)	k_a (x $10^5 M^{-1}s^{-1}$)	k_d (x $10^{-3} s^{-1}$)	K_D (nM)	N
**h4-1BB_wt**	37	14 ± 0.97	94 ± 2.2	69 ± 5.2	3
**h4-1BB-N42R**	37	11 ± 0.90	98 ± 13	92 ± 14	3
**h4-1BB-I132R**	37	n/d	n/d	barely binds at 500 nM	3
**h4-1BB-M101R**	37	n/d	n/d	barely binds at 500 nM	3

B. **Sensorgrams for h4-1BB receptor (WT and mutants) binding to Urelumab hIgG4 at 37°C**

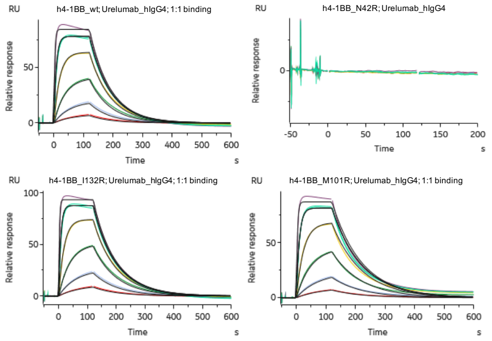

Binding kinetics and affinities of h4-1BB receptor (WT and mutants) to Urelumab hIgG4 at 37°C

Sample	T (°C)	k_a (x $10^5 M^{-1}s^{-1}$)	k_d (x $10^{-3} s^{-1}$)	K_D (nM)	N
**h4-1BB_wt**	37	6.3 ± 0.55	14 ± 1.1	22 ± 2.6	3
**h4-1BB-N42R**	37	n/d	n/d	no binding at 500 nM	3
**h4-1BB-I132R**	37	7.3 ± 0.66	12 ± 1.3	16 ± 2.4	3
**h4-1BB-M101R**	37	5.4 ± 0.27	9.5 ± 0.43	18 ± 1.2	3

Supplementary Figure 8: Binding and kinetics of wild type and mutant h4-1BB receptor to antibodies validating utomilumab and urelumab binding sites. Sensorgrams for h4-1BB receptor (WT and N42R, M101R and I132R mutants) binding to (A) Utomilumab hIgG2 or (B) Urelumab hIgG4 at 37°C. Triplicate measurements are consistent with one another, therefore, one replicate is shown. Results from kinetic analysis with a 1:1 langmuir model with mass transport of replicates summarized in tables (below).

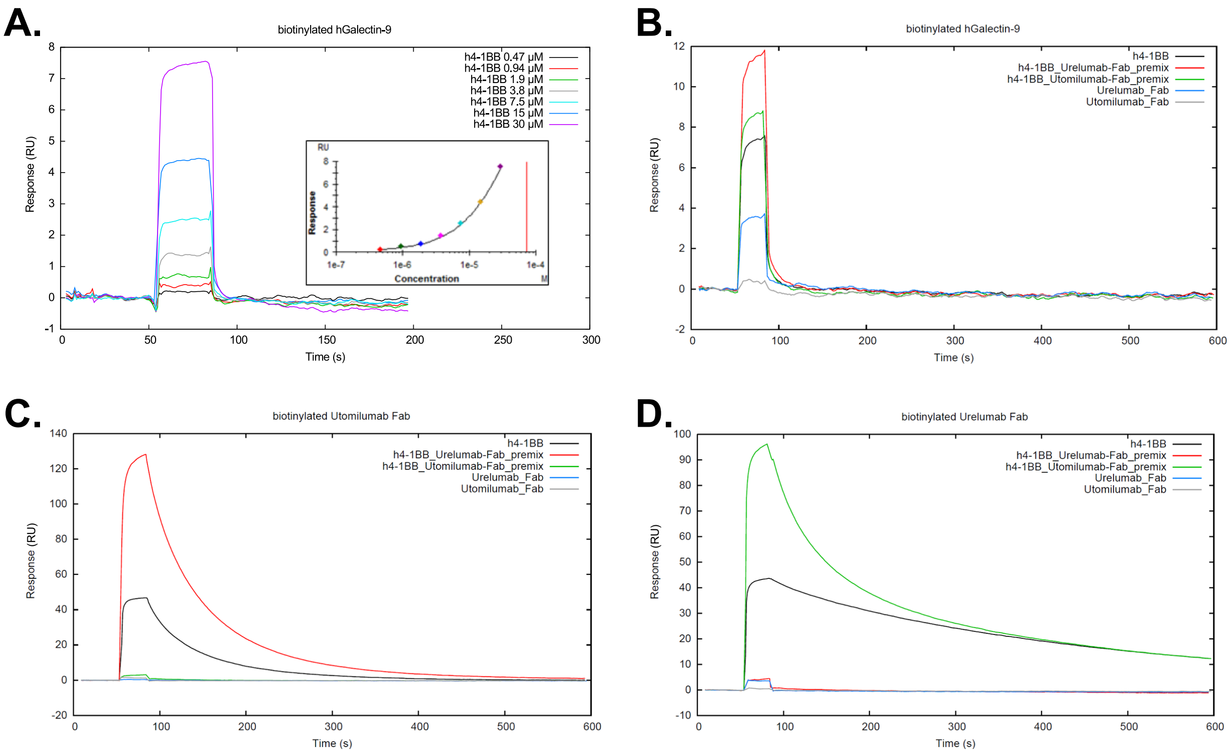

Supplementary Figure 9. Binding of h4-1BB to Galectin-9. **A)** Soluble h4-BB shows a dose response binding and a weak affinity to human Galectin-9 at 25°C. K_D is estimated to be greater than 15 μM. Biotinylated hGalectin-9 captured onto the streptavidin sensor with soluble monomeric h4-1BB ECD flowed as the analyze. Triplicate measurements were similar; therefore, only one replicate shown. The legend shows the concentration tested for h4-1BB. Double-referenced sensorgrams for the premix assay with: (**B**) biotinylated hGalectin-9; (**C**) biotinylated Utomilumab Fab; and (**D**) Urelumab Fab captured onto the streptavidin sensor surface. The legend shows binding of h4-1BB at 10 μM and premix mixtures of h4-1BB at 10 μM and Utomilumab Fab or Urelumab Fab at 20 μM. Both (**C**) and (**D**) were controls showing that Utomilumab and Urelumab Fabs self-block and sandwich with each other. Triplicate measurements are consistent with one another; therefore, only one replicate is shown.

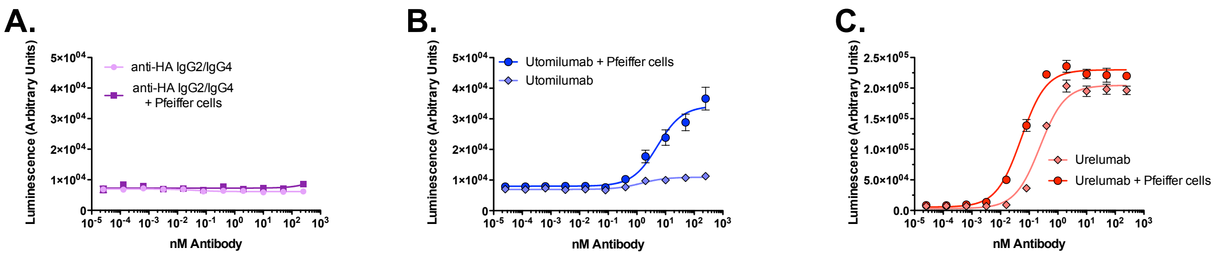

Supplementary Figure 10. Utomilumab and urelumab signaling activity in h4-1BB transduced NF-KB luciferaser reporter Jurkat cells +/- Pfeiffer cells or +/- 4-1BBL. Reporter cells were incubated +/- Pfeiffer cells with five-fold increasing concentrations (max 250 nM) of (**A**) negative control mixture of anti-HA hIgG2 and hIgG4 or (**B**) utomilumab antibody or (**C**) urelumab antibody at 37°C for 6hrs before measuring luciferase activity. Mean luminescence signal was plotted against concentrations of test antibodies. Error bars represent the standard deviation (SD) of five measurements for antibody alone and three measurements for antibody plus Pfeiffer cells and solid lines show fits with a standard dose-response curve.

Supplementary Table 1: Data collection and refinement statistics (molecular replacement)

	4-1BBL trimer	4-1BB / 4-1BBL complex	4-1BB / Utomilumab Fab complex	4-1BB / Urelumab Fab complex
**Data collection**				
Space group	P 62	C 2 2 2_1	I 1 2 1	C 2 2 2_1
Cell dimensions				
a, *b*, *c* (Å)	118.91, 118.91, 105.49	102.01, 229.38, 114.9	136.90, 73.34, 175.12	77.15, 192.06, 344.58
α, β, γ (º)	90, 90, 120	90, 90, 90	90, 98.99, 90	90, 90, 90
Resolution (Å)	46.95 - 2.95 (3.056 - 2.95)	48.91 - 2.13 (2.206 - 2.13)	49.3 - 2.72 (2.817 - 2.72)	49.65 - 2.8 (2.9 - 2.8)
R_{sym} or R_{merge}	0.2012 (3.471)	0.2039 (8.352)	0.350 (4.509)	0.2219 (4.068)
Mean I / σI	13.25 (0.70)	18.41 (0.64)	6.8 (0.5)	17.20 (0.89)
Completeness (%)	99.77 (99.05)	98.85 (95.49)	99.44 (95.61)	99.96 (99.94)
Redundancy	10.3 (10.2)	27.2 (27.7)	6.9 (7.0)	26.7 (27.9)
CC 1/2	0.997 (0.34)	0.999 (0.314)	0.985 (0.235)	0.998 (0.326)
**Refinement**				
Resolution (Å)	2.95	2.13	2.72	2.8
No. reflections	17937 (1796)	75057 (7363)	46170 (4379)	63532 (6235)
R_{work} / R_{free}	0.1948 (0.3698) / 0.2481 (0.4149)	0.2171 (0.4019) / 0.2514 (0.4049)	0.2393 (0.4020) / 27.73 (0.4312)	0.1964 (0.3924) / 0.2214 (0.4067)
No. atoms				
Protein	3298	6441	7881	8450
Ligand/ion	31	188	192	152
Water	16	88	46	15
B-factors				
Protein	95.05	69.87	86.24	78.93
Ligand/ion	154.66	112.05	113.65	111.25
Water	76.34	59.78	51.22	73.27
R.m.s. deviations				
Bond lengths (Å)	0.003	0.006	0.002	0.011
Bond angles (º)	0.65	0.89	0.63	1.31
PDB code	6MGE	6MGP	6MI2	6MHR

*Values in parentheses are for highest-resolution shell.

Supplementary Table 2

**Utomilumab heavy chain variable**	**EVQLVQSGAEVKKPGESLRISCKGSGYSFS TYWISWVRQMPGKGLEWMGKIYPGDSYTN YSPSFQGQVTISADKSISTAYLQWSSLKASD TAMYYCARGYGIFDYWGQGTLVTVSS**
	**gaagtccagcttgtccagtcaggagcagaagtgaa aaagcccggcgaatccctgagaatttcctgcaagg gctccgggtactccttctcgacctattggattagctg ggtgcgccagatgcctgggaagggtctggagtgg atgggaaagatctacccaggagactcgtacaccaa ctactccccgtcattccaaggacaagtcaccatctc cgccgacaagtctatctccactgcgtacctccagtg gagcagcctgaaggcctcggatactgccatgtact actgtgctcggggttacggcatctttgactactgggg acagggcaccctggtcaccgtctcctca**
**Utomilumab Lambda chain variable**	**SYELTQPPSVSVSPGQTASITCSGDNIGDQY AHWYQQKPGQSPVLVIYQDKNRPSGIPERF SGSNSGNTATLTISGTQAMDEADYYCATYTG FGSLAVFGGGTKLTVL**
	**tcctacgaacttacccagccgccatcagtcagcgtgtccc ccggccaaactgcttccatcacgtgctccggagacaaca ttggggatcagtacgcccactggtatcagcagaagcctg gacagtcacccgtgctcgtgatctaccaggataagaatc gcccgagcggaatccctgaacggttttccgggagcaact cgggaaacaccgccaccctgaccatttccggtactcaag caatggacgaggccgactactactgtgccacctacaccg gcttcggttcgctggcggtgttcggcggaggcaccaagc tgactgtgcta**

Supplementary Methods

SPR analysis of wild type and mutant h4-1BB receptor binding to wild type h4-1BBL

Binding of human 4-1BB wild type and mutant (I64R and V71R) receptors to h4-1BBL wild type was determined on a Biacore 8K SPR instrument (GE Lifesciences, Marlborough, MA) at 37°C in running buffer HBS supplemented with 0.05% (v/v) Tween-20 and 1 mg/mL BSA.

A Biacore Series S sensor chip CAP (catalog No. 28920234, GE Lifesciences, Marlborough, MA) was rehydrated with water at 42°C for 1 hour and then dried with

compressed air. The surface of the chip was conditioned with three, 1 minute injections of a mixture of 3 parts of 8 M guanidine-HCl with 1 part 1M NaOH. Biotin CAPture reagent was injected for 5 minutes at 2 µL/min. Biotinylated h4-1BBL wild-type at 10 µg/mL or buffer was captured for 2 minutes at 10 µL/min. Then, surfaces were blocked with 20 µM amine-PEG_2-biotin (catalog No. 21346, ThermoFisher Scientific, Waltham, MA) for 1 minute at 10 µL/min. h4-1BB-wt, h4-1BB-I64R, and h4-1BB-V71R were diluted to 300 nM into running buffer. h4-1BB-wt, h4-1BB-I64R, h4-1BB-V71R, or buffer was injected as analyte for 2 minutes at 30 µL/min and dissociation monitored for 10 minutes. The sensor chip CAP surfaces were regenerated for 2 minutes at 10 µL/min with a mixture of 3 parts of 8 M guanidine-HCl with 1 part 1M NaOH between each analyte binding cycle. After regeneration of the surfaces, all parts of the flow system were washed with buffer. The experiment was run in triplicates. All sensorgrams were double-referenced [69].

SPR analysis of wild type and mutant h4-1BB receptor binding to utomilumab hIgG2 and urelumab hIgG4

The kinetics and affinity for human 4-1BB wild type and mutant receptors binding to utomilumab hIgG2 and urelumab hIgG4 were determined on a Biacore 8K SPR instrument (GE Lifesciences, Marlborough, MA).

An anti-human Fc capture chip was prepared by amine-coupling goat anti-human IgG Fc (catalog No. 2014-01, SouthernBiotech, Birmingham, AL) to a Biacore Series S sensor chip CM4 (catalog No. BR100534, GE Lifesciences, Marlborough, MA) at 25°C. The running buffer for the immobilization procedure was HBS with 0.05% (v/v) Tween-20. The following was performed in all flow cells simultaneously to result in all surfaces containing amine-coupled anti-human IgG Fc. The CM4 chip surface was activated with 1:1 (v/v) mixture of 400 mM 1-Ethyl-3-(3-Dimethylaminopropyl) carbodiimide hydrochloride (EDC) and 100 mM N-Hydroxysuccinimide (NHS) for 7 minutes at 10 µL/min followed by washing all parts of the flow system except the sensor

chip flow cells with 100 mM ethylenediamine in 200 mM borate buffer pH 8.5. The anti-human IgG Fc was diluted to 50 µg/mL in 10 mM sodium acetate pH 4.5 and injected at 10 µL/min for 7 minutes. Excess reactive esters on the surface were blocked for 7 minutes at 10 µL/min with 100 mM ethylenediamine in 200 mM borate buffer pH 8.5. After immobilization, the surface was conditioned with three 1-minute injections of 75 mM phosphoric acid at 10 µL/min.

Kinetic assays were performed at 37°C in running buffer HBS supplemented with 0.05% (v/v) Tween-20 and 1 mg/mL BSA. Utomilumab hIgG2, urelumab hIgG2, and 4FNL-hIgG1 (an anti-hemagglutinin antibody as negative control) were diluted to 10 µg/mL into running buffer and captured onto anti-human Fc surfaces for 2 minutes with a flow rate of 10 µL/min. h4-1BB-wt, h4-1BB-I132R, h4-1BB-N42R and h4-1BB-M101R were diluted into running buffer and a serial dilution series was created with a 3-fold dilution factor to give concentrations of 2.1, 6.2, 18.5, 55.6, 166.7, and 500 nM. h4-1BB-wt, h4-1BB-I132R, h4-1BB-N42R, h4-1BB-M101R or buffer was injected as analyte for 2 minutes at 30 µL/min and dissociation monitored for 15 minutes. The 166.7 nM samples were run in duplicate and all interactions were investigated in triplicate using 3 independent analyte dilution series. The anti-human Fc surfaces were regenerated with three 60-second injections of 75 mM phosphoric acid at 10 µL/min between each analyte binding cycle.

All sensorgrams were double-referenced [69] and fit to a 1:1 Langmuir binding model with mass transport using Biacore 8K Evaluation Software (version 1.1.1.7442).

SPR analysis of human 4-1BB receptor binding to human galectin-9

The affinity for the interaction of human 4-1BB receptor with human galectin-9 was determined on a Biacore T200 SPR instrument (GE Lifesciences, Marlborough, MA) at 25°C.

Human galectin-9 (catalog No. 2045-GA, R&D Systems, Minneapolis, MN) was biotinylated with a 1:1 molar ratio of sulfo-NHS-LC-LC-biotin (catalog No. 21388,

ThermoFisher Scientific, Waltham, MA) to protein, and buffer exchanged into PBS using ThermoFisher Zeba 7K MWCO columns (catalog No. 89883, ThermoFisher Scientific, Waltham, MA).

The running and dilution buffer for the immobilization procedure was HBS supplemented with 0.05% Tween-20. A Biacore Streptavidin Series S sensor chip (catalog No. BR100398, GE Lifesciences, Marlborough, MA) was preconditioned with 3, 60 second injections of a mixture of 50 mM NaOH – 1 M NaCl at 10 µL/min. Biotinylated human galectin-9 at 10 µg/mL was injected for 5 min at 20 µL/min in flow cell 2. Blank immobilization in flow cell 1 as reference surface. After captured of the biotinylated human galectin-9, surfaces in flow cells 1 and 2 of the Streptavidin sensor chip were blocked with 20 µM amine-PEG_2-biotin (catalog No. 21346, ThermoFisher Scientific, Waltham, MA) at 10 µL/min for 60 second. Final immobilization level for biotinylated human galectin-9 was 1101 RU.

Kinetic assay was conducted with HBS supplemented with 0.05% Tween-20 and 1 mg/mL BSA as running and dilution buffer. Human 4-1BB at concentrations of 0.47, 0.94, 1.9, 3.8, 7.5, 15, and 30 µM, or buffer were injected as analytes at a flow rate of 30 µL/min for 30 seconds and dissociation was monitored for 3 minutes. The interaction was measured in triplicate using three independent analyte dilution series. Human 4-1BB was allowed to dissociate to baseline before measuring replicates.

Blocking assay of the interaction of human 4-1BB receptor with human galectin-9 by utomilumab Fab or urelumab Fab

Blocking of the interaction of human 4-1BB receptor with human galectin-9 by utomilumab Fab or urelumab Fab was performed in a premix assay format on a Biacore T200 SPR instrument (GE Lifesciences, Marlborough, MA) at 25°C.

Utomilumab Fab and urelumab Fab were biotinylated with a 1:1 molar ratio of sulfo-NHS-LC-LC-biotin (catalog No. 21388, ThermoFisher Scientific, Waltham, MA) to Fab,

and buffer exchanged into PBS using ThermoFisher Zeba 7K MWCO columns (catalog No. 89883, ThermoFisher Scientific, Waltham, MA).

The running and dilution buffer for the immobilization procedure was HBS supplemented with 0.05% Tween-20. A Biacore Streptavidin Series S sensor chip (catalog No. BR100398, GE Lifesciences, Marlborough, MA) was preconditioned with 3, 60 second injections of a mixture of 50 mM NaOH – 1 M NaCl at 10 µL/min. In flow cell 2, human galectin-9, biotinylated as described previously, was injected at 10 µg/mL for 5 min at 20 µL/min. In flow cell 3, biotinylated utomilumab Fab was injected at 0.1 µg/mL for 40 seconds at 20 µL/min followed by a 30 second injection at 1 µg/mL. In flow cell 4, biotinylated urelumab Fab was injected at 1 µg/mL for 20 second at 20 µL/min. In flow cell 1, blank immobilization as reference surface. After immobilization of the biotinylated ligands, surfaces in all flow cells were blocked with 20 µM amine-PEG_2-biotin (catalog No. 21346, ThermoFisher Scientific, Waltham, MA) at 10 µL/min for 60 second. Final immobilization levels for biotinylated human galectin-9, biotinylated utomilumab Fab, and biotinylated urelumab Fab were 1101 RU, 172 RU, and 90 RU, respectively.

The running and dilution buffer for the premix assay was HBS supplemented with 0.05% Tween-20 and 1 mg/mL BSA. Buffer, utomilumab Fab or urelumab Fab at 20 µM, h4-1BB at 10 µM, or a premix mixture of h4-1BB at 10 µM and utomilumab Fab or urelumab Fab at 20 µM was injected as analyte in all flow cells at 30 µL/min for 30 seconds followed by 30 minute dissociation. The interactions were allowed to dissociate to baseline before measuring replicates. The experiment was run in triplicate.
